# Modularly assembled multiplex prime editors for simultaneous editing of agronomically important genes in rice

**DOI:** 10.1016/j.xplc.2023.100741

**Published:** 2023-10-26

**Authors:** Ajay Gupta, Bo Liu, Saad Raza, Qi-Jun Chen, Bing Yang

**Affiliations:** 1 Division of Plant Science and Technology, Bond Life Sciences Center, University of Missouri, Columbia, MO 65211, USA; 2 State Key Laboratory of Plant Physiology and Biochemistry, College of Biological Sciences, China Agricultural University, Beijing 100193, China; 3 Center for Crop Functional Genomics and Molecular Breeding, China Agricultural University, Beijing 100193, China; 4 Donald Danforth Plant Science Center, St. Louis, MO 63132, USA

**Keywords:** prime editing, rice, bacterial blight, herbicide tolerance, multiplex genome editing

## Abstract

Prime editing (PE) technology enables precise alterations in the genetic code of a genome of interest. PE offers great potential for identifying major agronomically important genes in plants and editing them into superior variants, ideally targeting multiple loci simultaneously to realize the collective effects of the edits. Here, we report the development of a modular assembly-based multiplex PE system in rice and demonstrate its efficacy in editing up to four genes in a single transformation experiment. The duplex PE (DPE) system achieved a co-editing efficiency of 46.1% in the T_0_ generation, converting *TFIIAγ5* to *xa5* and *xa23* to *Xa23*^*SW11*^. The resulting double-mutant lines exhibited robust broad-spectrum resistance against multiple *Xanthomonas oryzae* pathovar *oryzae* (*Xoo*) strains in the T_1_ generation. In addition, we successfully edited *OsEPSPS1* to an herbicide-tolerant variant and *OsSWEET11a* to a *Xoo*-resistant allele, achieving a co-editing rate of 57.14%. Furthermore, with the quadruple PE (QPE) system, we edited four genes—two for herbicide tolerance (*OsEPSPS1* and *OsALS1*) and two for *Xoo* resistance (*TFIIAγ5* and *OsSWEET11a*)—using one construct, with a co-editing efficiency of 43.5% for all four genes in the T_0_ generation. We performed multiplex PE using five more constructs, including two for triplex PE (TPE) and three for QPE, each targeting a different set of genes. The editing rates were dependent on the activity of pegRNA and/or ngRNA. For instance, optimization of ngRNA increased the PE rates for one of the targets (*OsSPL13*) from 0% to 30% but did not improve editing at another target (*OsGS2*). Overall, our modular assembly-based system yielded high PE rates and streamlined the cloning of PE reagents, making it feasible for more labs to utilize PE for their editing experiments. These findings have significant implications for advancing gene editing techniques in plants and may pave the way for future agricultural applications.

## Teaser

This study reports the development of modularly assembled multiplex prime editors and their capacity to simultaneously target up to four genomic loci at high efficiency in rice. Multiplex prime editing can result in rice lines carrying gene copies that confer broad resistance to bacterial blight and strong tolerance to herbicides.

## Introduction

Simultaneously creating multiple genetic variations and breeding them into improved germplasm is the most desirable objective of any crop improvement program. It is necessary to target and modify multiple genes at once to harness the additive benefits of multiple genic combinations in a genotype of interest (Chen et al., 2019). Double-stranded DNA-break-inducing CRISPR systems, which are mainly used to generate insertion/deletion-type knockout mutants, can rarely generate superior alleles, which require precise genomic changes. Prime editing (PE), on the other hand, has broader applications because of its ability to generate DNA insertions and deletions as well as install desired base-pair changes (Anzalone et al., 2019). PE is the latest and most advanced CRISPR-based genome editing technology and has been revolutionizing biology by enabling scientists to search for and replace genomic sequences without the need for a double-stranded DNA break and a donor template carrying the desired sequence changes. In brief, a reverse transcriptase-fused Cas9 nickase (nCas9–RT) targets the genomic region guided by a PE RNA (pegRNA). The pegRNA directs the nCas9–RT/pegRNA complex to the target region; the edit encoded in the 3′ extension is reverse transcribed to the 3′ end of the nicked genomic DNA strand. This leads to the generation of a 3′ flap containing the edited sequence and a 5′ flap of wild-type (WT) sequence surrounding the nicked site. These flaps are resolved via a flap excision and heteroduplex repair system. Excision of the 5′ flap and incorporation of the 3′ flap lead to an editing event (Anzalone et al., 2019). A second guide RNA (nicking guide RNA; ngRNA) is used to nick the opposite strand either upstream or downstream of the original target to render the excision repair in favor of 3′ flap incorporation (Anzalone et al., 2019; Yang et al., 2019). Until recently, PE has suffered from low editing efficiency in plants, but with the new ePPE, PE3max, and PE5max systems, editing efficiencies have been boosted up to 88% for some single-site targets in rice (Anzalone et al., 2019; Li et al., 2022b; Jiang et al., 2022; Gupta et al., 2023b). This high efficiency makes the PE system amenable to multiplexing.

PE can provide an unprecedented opportunity, ideally, to target several heterologous genes or multiple sites of single genes simultaneously and install multiple desired changes in a single transformation event, including knockouts, small insertions/deletions, and specific base pair changes (Yang et al., 2019; Molla et al., 2021). This enables the simultaneous improvement of several agronomically important traits in crop plants. For instance, yield-related genes can be targeted to improve crop productivity, disease-resistance genes can be stacked to provide broader and more durable resistance against numerous diseases, and abiotic stress-related genes can be edited to increase crop resilience in the face of a changing climate (Hassan et al., 2020; Molla et al., 2021; Gupta et al., 2023b; Li et al., 2023). Furthermore, these edits in multiple traits can be combined to engineer new germplasm with improved productivity and enhanced resilience to biotic and abiotic stresses. Moreover, the multiplex PE system can serve as a valuable asset for functional genomics by enabling researchers to change the native gene sequence instead of relying on complementation assays with a transgenic approach. Multiple protein tagging with PE can help us understand complex gene-regulatory networks by tracking protein expression in native conditions (Hua et al., 2022; Kumar et al., 2023).

Rice is an important staple crop in terms of global food security, and it also serves as the model crop for studying cereal genetics and developing genetic tools. It is highly desirable to have a high-efficiency multiplex PE system in rice to facilitate rice crop improvement programs and study the functions of agronomically important genes/traits in an endogenous context (Huang and Puchta, 2021; Xu et al., 2022; Ni et al., 2023). Here, we report the development of a modular PE system amenable to multiplexing pegRNAs together with ngRNAs and editing up to four genomic loci. The modular assembly uses Golden Gate cloning to make individual pegRNA–ngRNA units and subsequently uses Gateway recombination to combine the pegRNA–ngRNA units with the final nCas9–RT vector. We validated the feasibility and efficacy of this system by targeting two, three, or four genes in a single generation, approaches named duplex PE (DPE), triplex PE (TPE), or quadruplex PE (QPE), respectively. In our multiplexing experiments, we achieved high editing efficiencies, with a number of lines carrying simultaneous monoallelic and biallelic edits in the T_0_ generation. We also obtained lines with both genes edited by DPE, all three genes edited by TPE, and all four genes edited by QPE. In this work, we simultaneously edited genes related to bacterial blight of rice, herbicide tolerance, plant architecture, and grain yield, demonstrating the phenotypic superiority of the edited lines over the unedited lines in terms of bacterial blight resistance and herbicide tolerance.

## Results

### Modular assembly of multiplex prime editing constructs

**Figure 1:**
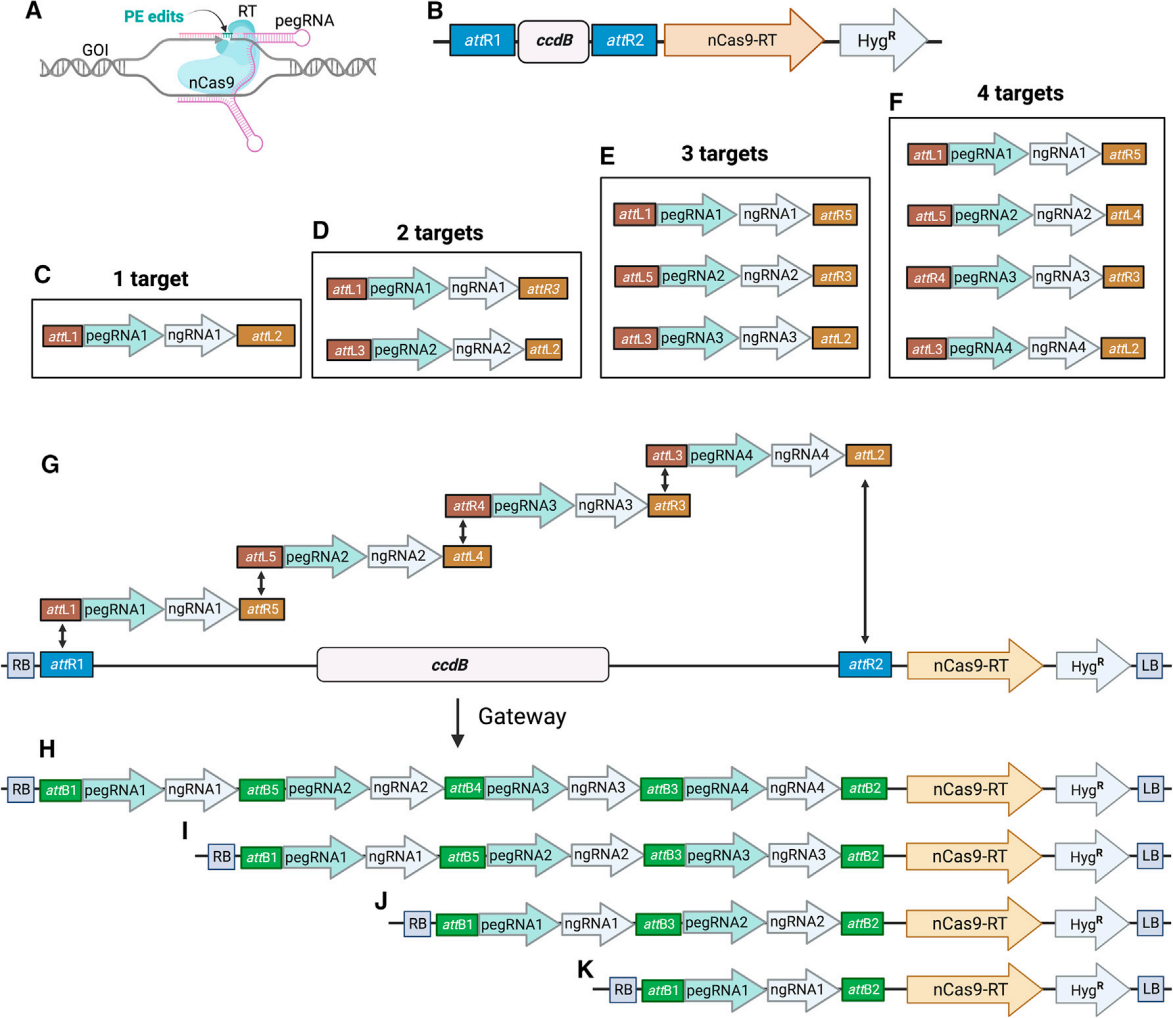
Modular assembly of multiplex prime editing constructs. **(A)** Schematic representation of the required components for prime editing. **(B)** Binary vector carrying nickase Cas9 fused with reverse transcriptase, the hygromycin-resistance gene, and a Gateway cassette to accept inserts from entry clones. **(C–F)** Entry vectors for cloning up to four pegRNA–ngRNA units flanked by variable attachment (att) regions. **(G)** Schematic of the Gateway reaction transferring four pegRNA–ngRNA cassettes from entry clones to the destination vector. **(H–K)** Resulting final vectors ready for *Agrobacterium*/bombardment-mediated plant transformation for prime editing of up to four genes.

The core requirements of PE3 include a nicking Cas9 (H840A) fused to a reverse transcriptase (nCas9–RT), a pegRNA that consists of a single guide RNA with a spacer specifying the target and a reverse transcription template (rtT) encoding information about the edit as well as a prime binding sequence (PBS), and an ngRNA to nick the opposite strand to favor DNA repair toward the edited strand (Figure 1A) (Anzalone et al., 2019). The rtT is located downstream of the pegRNA scaffold followed by the PBS. A nuclease-resistant RNA motif, evopreQ1, is used at the 3′ end to prevent RNA degradation. PBS and evopreQ1 are separated by an 8-bp linker calculated using the webtool pegLIT (Supplemental Protocol). The paired pegRNA–ngRNA format (namely, PE3 or PE3b) is an improvement on the initial PE2 (Anzalone et al., 2019; Yang et al., 2019). Because nCas9–RT is the constant reagent for different PE events, whereas the pegRNA and ngRNA change with every new target, we designed a modular assembly-based PE system for easy cloning of multiple pegRNA–ngRNA units (Figure 1A and 1B). In this modular system, the nCas9–RT is included in a destination binary vector that can accept multiple pegRNA–ngRNA units using the Gateway cloning approach. To construct individual pegRNA–ngRNA units, multiple entry vectors flanked by different Gateway recombination sequences (RS, *att*L, and *att*R) were designed and constructed.

These Gateway RSs are different combinations of *att*L and *att*R sequences that enable the assembly of multiple pegRNA–ngRNA units into a single destination vector. For single pegRNA–ngRNA assembly, *att*L1–*att*L2 was used to flank the pegRNA–ngRNA cassette; *att*L1–*att*R3 and *att*L3–*att*L2 were used for two pegRNA–ngRNA units; *att*L1–*att*R5, *att*L5–*att*R3, and *att*L3–*att*L2 for three pegRNA–ngRNA units; and *att*L1–*att*R5, *att*L5–*att*L4, *att*R4–*att*R3, and *att*L3–*att*L2 for four pegRNA–ngRNA units (Figure 1C–1F, Supplemental Sequences 1–11). These entry vectors were designed to insert a spacer sequence for pegRNA at the *BsmB*I site and rtT-PBS and a spacer sequence for ngRNA at the *Bsa*I site using the Golden Gate procedure or a regular restriction–ligation protocol (Supplemental Protocol). All pegRNA spacer, rtT-PBS, and ngRNA spacer sequences can be derived from respective complementary oligos forming double-stranded fragments with appropriate 5′ overhangs. After cloning of the pegRNA–ngRNA units, up to four units can be mobilized into a single binary vector at the *att*R1–*att*R2 site using Gateway cloning (Supplemental Protocol). Using this approach, we successfully assembled one, two, three, and four units and confirmed the cloning via whole-plasmid sequencing (Figure 1G–1K). These constructs remained stable in *Agrobacterium* and the transfer-DNA transfer of these units was also found to be stable in rice cells. In this approach, expression of each pegRNA–ngRNA unit is driven by an individual promoter, ensuring high and uniform expression of each unit. We used this assembly to design and construct all the pegRNA–ngRNA units used for this study.

### Duplex prime editing efficiently generates *Xa23*^*SW11*^ and *xa5* co-edited lines in the T_0_ generation

**Figure 2:**
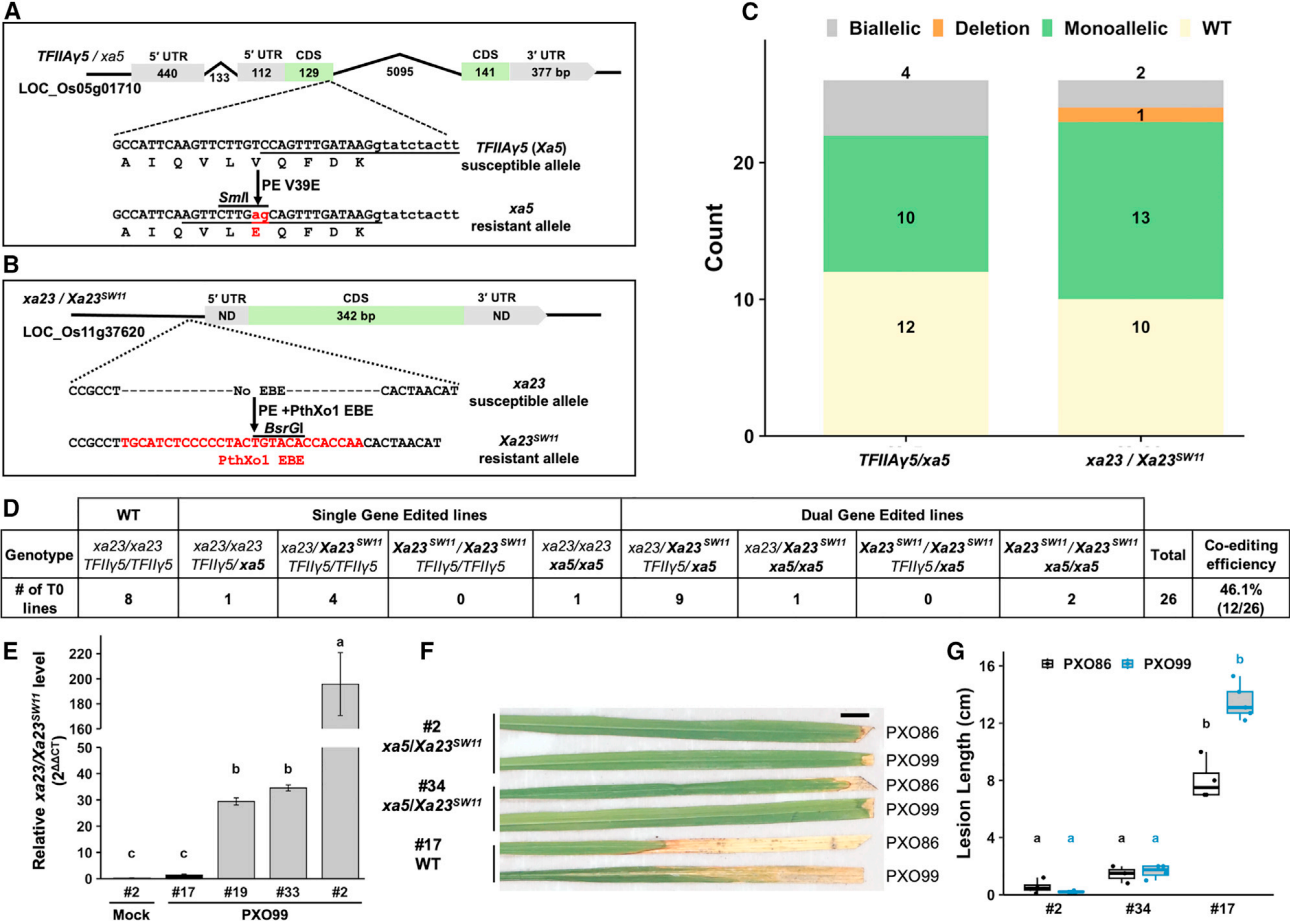
Duplex prime editing of *Xa23*^*SW11*^ and *xa5* lines. **(A)** Gene structures of *TFIIAγ5* and *xa5*. The PE target site (underlined) in the *TFIIAγ5* allele and the nick site (underlined) in the edited strand of the *xa5* allele are shown. **(B)** Gene structures of *xa23* and *Xa23*^*SW11*^. The intronless coding sequence (CDS) and untranslated sequences (ND, not determined) are shown. **(C)** Counts of monoallelic, biallelic, and WT lines based on PCR-RE-based genotyping of *TFIIAγ5/xa5*-edited lines with *Sml*I digestion of relevant PCR amplicons and *xa23*/*Xa23*^*SW11*^*-*edited lines with *Bsr*GI digestion of relevant PCR amplicons. Numbers of monoallelic, biallelic, deletion, and WT lines are mentioned in each box. **(D)** Summary of genotyping based on PCR-RE. Edited alleles are mentioned in bold. **(E)** qRT-PCR of the *xa23/Xa23*^*SW11*^ gene. All samples were normalized against *OsActin* (housekeeping gene control), and the comparison was made against an unedited line infiltrated with PXO99. **(F and G)** Disease phenotypes of edited biallelic lines. Lines #2 and #34 are biallelic for *xa5* and *Xa23*^*SW11*^ alleles. Lesion lengths were measured 12 days post inoculation with PXO86 and PXO99 on three to five leaves of individual plants (n = 3–5). Scale bar, 1 cm. Lowercase letters a, b, and c in **(E)** and letters a and b in **(G)** represent statistically significant differences among different treatments calculated by Tukey’s test.

In our previous study (Gupta et al., 2023b), *xa5*-edited rice lines exhibited strong broad-spectrum resistance against all *Xoo* strains dependent on *TFIIAγ5* for *SWEET* gene induction by major transcription activator-like effectors (TALEs). However, *Xoo* strains carrying the TALE gene *pthXo1* can overcome *xa5*-mediated resistance, as *pthXo1* can use both *TFIIAγ5* and *xa5* efficiently for *SWEET11a* induction (Huang et al., 2016). Therefore, we decided to utilize the DPE system, with as-yet unknown efficiency, to perform duplex editing of V39E substitution in *TFIIAγ5* to generate the *xa5* allele and, by inserting the 28-bp-long PthXo1 effector-binding element (EBE) of *OsSWEET11a* into the promoter of dysfunctional *xa23*, to generate a functional *Xa23*^*SW11*^ allele variant. The DPE construct encoding *TFIIAγ5* to *xa5* and *xa23* to *Xa23*^*SW11*^ was used for *Agrobacterium*-mediated rice transformation (Figure 2A and 2B). Twenty-six independent transgenic events were recovered and genotyped for the V39E edit based on the *Sml*I restriction sequence in *TFIIAγ5*/*xa5* and the PthXo1 EBE knockin based on the *BsrG*I restriction sequence that arose from successful editing in *xa23*/*Xa23*^*SW11*^ (Figure 2C and Supplemental Figure 1A and 1B). Of 26 T_0_ lines, 18 contained the edits for *xa5*, *Xa23*^*SW11*^, or both, resulting in an editing efficiency of 69.23% (Figure 2C and 2D and Supplemental Figure 1A and 1B; Supplemental Table 3). Of the 18 edited lines, 12 lines were co-edited for both *xa5* and *Xa23*^*SW11*^, leading to a co-editing efficiency of 46.1% (Figure 2C and 2D and Supplemental Figure 1A and 1B; Supplemental Table 3). Two of these 12 lines were double biallelic (both alleles of *TFIIAγ5* and *xa23* edited), 9 were double monoallelic (one allele each of *TFIIAγ5* and *xa23* edited), and 1 was biallelic for *TFIIAγ5* and monoallelic for *xa23* (Figure 2C and 2D and Supplemental Figure 1A and 1B; Supplemental Table 3). To validate the accuracy of editing, we deep sequenced the amplicons from several lines of both *xa5-* and *Xa23*^*SW11*^-edited lines (Supplemental Figure 2A and 2B). For biallelic lines, more than 85% of reads mapped to an edited allele, and for monoallelic lines, >40% of reads out of the total mapped to an edited allele (Supplemental Figure 2A and 2B). We further validated these results by Sanger sequencing of two double-biallelic lines (#2 and #34) (Supplemental Figure 2C and 2D). Sequencing chromatograms also revealed that lines #2 and #34 were biallelic for both *xa5* and *Xa23*^*SW11*^ (Supplemental Figure 2C and 2D). These results indicate the feasibility of the highly efficient PE3max system for DPE. This is the first report to generate these two loci in the same genetic background, making it vital to test the activity of both loci, especially *Xa23*^*SW11*^, in the same genetic background*.* The *xa5* allele is known to be less effective at enabling *pthXo1*-mediated induction of *SWEET11a* (Huang et al., 2016). Thus, to test the induction of *Xa23*^*SW11*^ in the *xa5* background, we infiltrated the PXO99 (*pthXo1*) and PXO86 (*avrXa7*) strains into the *Xa23*^*SW11*^/*xa5* dual-biallelic, *Xa23*^*SW11*^ monoallelic, and WT lines. Total RNA was extracted 24 h post inoculation, first-strand cDNA was synthesized, and both semi-quantitative RT-PCR and quantitative RT-PCR were performed on all samples with equal amounts of cDNA. The *Xa23*^*SW11*^/*xa5* dual-biallelic line (#2) showed the highest induction of the *Xa23*^*SW11*^ gene upon PXO99 infection, followed by the *Xa23*^*SW11*^ monoallelic lines (#19 and #33) (Figure 2E and Supplemental Figure 3). The WT line (#17), PXO86-inoculated edited lines, and uninoculated lines showed no induction of *Xa23*^*SW11*^, suggesting tight regulation and induction due only to presence of PthXo1-EBE (Figure 2E and Supplemental Figure 3). Meanwhile, no difference in *SWEET11a* gene induction due to PXO99 was observed in any dual-edited line compared with the WT line, again confirming that *xa5* has less effect on *SWEET11a* induction by PthXo1 (Supplemental Figure 3). Furthermore, we challenged the edited and WT lines with PXO86 and PXO99 using the leaf-clipping inoculation method to test resistance in the T_0_ generation. The dual-biallelic lines were highly resistant to both strains, the *Xa23*^*SW11*^ monoallelic lines were resistant only to PXO99, and the WT lines were susceptible to both strains (Figure 2F and 2G). These results indicate that both engineered loci can work in the same genetic background and could provide strong broad-spectrum resistance against multiple strains.

### *xa5/Xa23*^*SW11*^ dual-edited lines provide broad spectrum resistance to multiple strains in the T_1_ generation

**Figure 3:**
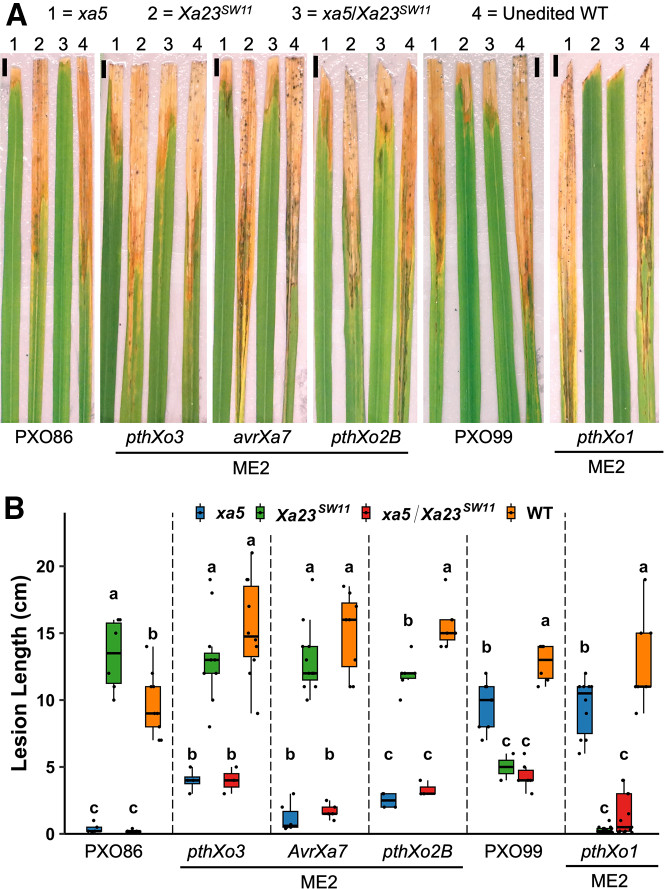
*xa5/Xa23*^*SW11*^ dual-edited lines exhibit broad-spectrum resistance to multiple strains in the T_1_ generation. **(A and B)** Disease phenotypes of edited homozygous T_1_ lines of *xa5*/*Xa23*^*SW11*^. The *Xoo* strains used for inoculation are indicated at the bottom. A numerical key is used to represent the different genotypes in **(A)**, and a color key is used for **(B)**. Lesion lengths were measured 12 days post inoculation on three to five leaves of individual plants (n = 3–5). Scale bar, 1 cm. In the bar graph, letters a, b, and c represent statistically significant differences in lesion lengths of edited and WT lines for all strains calculated by Tukey’s test.

We grew four T_0_ lines to test the heritability of *xa5/Xa23*^*SW11*^ dual- and single-edited lines in the T_1_ generation. All four lines carried the edits to the T_1_ generation, as confirmed by PCR and restriction enzyme digestion (PCR-RE) and Sanger sequencing. Next, we challenged these T_1_ lines with multiple *Xoo* strains carrying different TALEs to test the broad spectrum of resistance. Specifically, PXO86, ME2(*pthXo3*), ME2(*pthXo2B*), and ME2(*AvrXa7*) were selected for testing *xa5*-mediated resistance, and PXO99 and ME2(*pthXo1*) were selected for testing *Xa23*^*SW11*^-mediated resistance. The *xa5*-edited lines were highly resistant to strains PXO86, ME2(*pthXo3*), ME2(*pthXo2B*), and ME2(*AvrXa7*) but susceptible to PXO99 and ME2(*pthXo1*) (Figure 3A and 3B). By contrast, the *Xa23*^*SW11*^-edited lines were resistant only to PXO99 and ME2(*pthXo1*) and susceptible to PXO86, ME2(*pthXo3*), ME2(*pthXo2B*), and ME2(*avrxa7*) (Figure 3A and 3B). The duplex-edited lines carrying the *xa5/Xa23*^*SW11*^ genotype were resistant to all the tested strains. These results suggest that the two edits, *xa5* and *Xa23*^*SW11*^, can work synergistically to provide resistance against different TALE-carrying strains (Figure 3A and 3B).

### Duplex prime editing efficiently generates *EPSPS1 TAP-IVS* and *SWEET11a* EBE-deletion co-edited lines in the T_0_ generation

**Figure 4:**
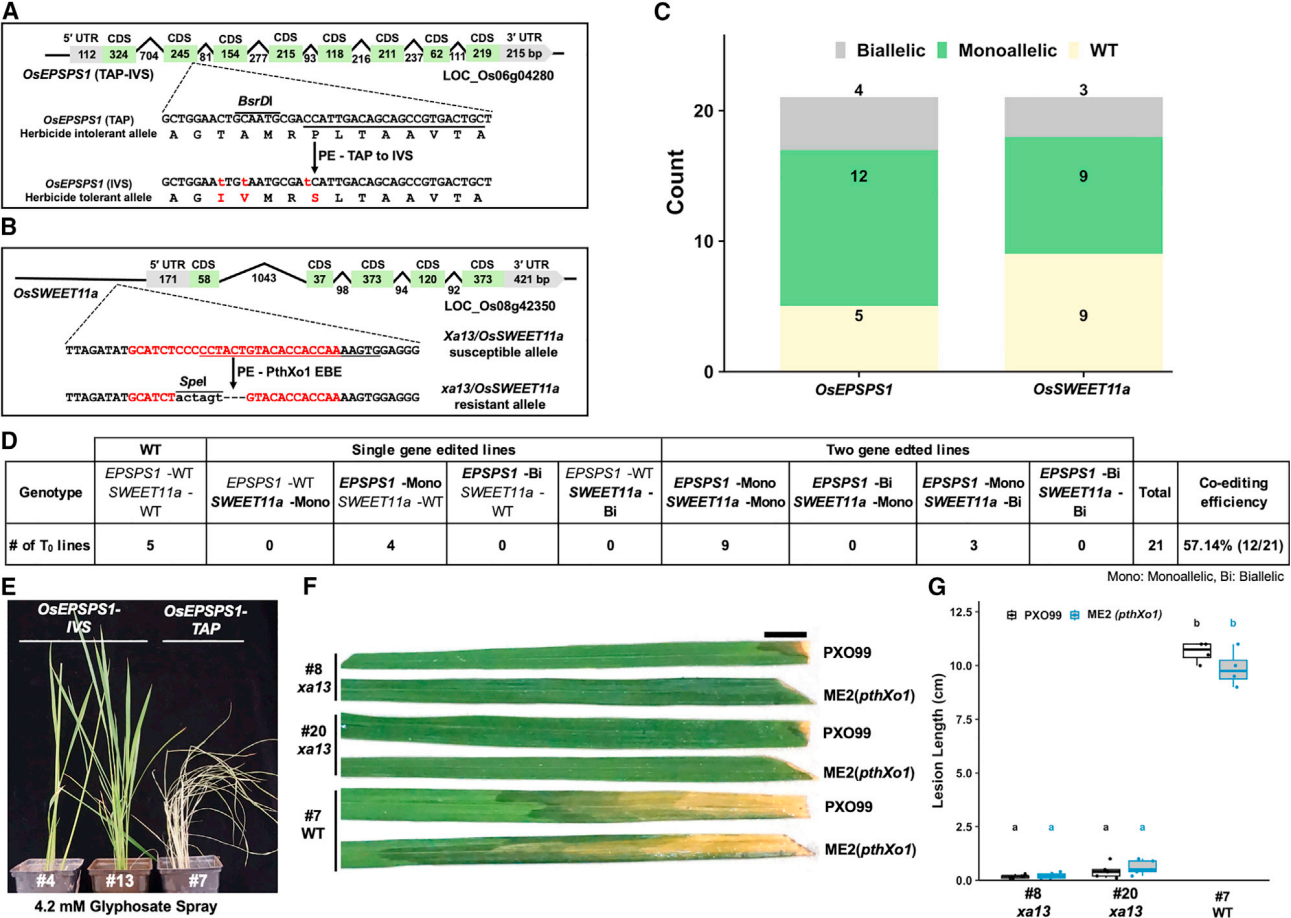
Duplex prime editing of *EPSPS1* for *TAP-IVS* editing and *SWEET11a* EBE deletion/knockout. **(A)** Gene structures of the *OsEPSPS1*-TAP allele and *OsEPSPS1*-IVS allele. The PE target site in the *OsEPSPS1*-TAP allele is underlined, and the desired amino acid change is shown in red in the *OsEPSPS1*-IVS allele. **(B)** Gene structures of *OsSWEET11a/xa13* and *xa13*. The effector binding element (EBE) in the promoter of susceptible *OsSWEET11a* is shown in red. The desired edit, including partial EBE deletion, and the *Spe*I recognition site insertion are shown in the resistant allele *xa13*. **(C)** Counts of monoallelic, biallelic, and WT lines based on genotyping of *OsEPSPS1*-edited lines with *BsrD*I digestion of relevant PCR amplicons and *OsSWEET11a/xa13*-edited lines with *Spe*I digestion of relevant PCR amplicons. **(D)** Summary of genotyping based on PCR-RE. Edited alleles are shown in bold. **(E)** Treatment of *OsEPSPS1*-edited and WT lines with 4.2 mM glyphosate spray. Genotypes are indicated at the top. Picture was taken 10 days after spraying. **(F and G)** Disease phenotypes of edited biallelic lines. Lesion lengths were measured 12 days post inoculation with PXO99 and ME2(*pthXo1*) on three to five leaves of individual plants (n = 3–5). Scale bar, 1 cm. Letters a and b in **(G)** represent statistically significant differences in lesion lengths of edited versus WT lines for both PXO99 and ME2(*pthXo1*) calculated by Tukey’s test.

Next, we tested DPE with another set of targets to generate the *OsEPSPS1* TAP-IVS triple amino acid substitution and *OsSWEET11a* EBE-deletion mutation. In *OsEPSPS1*, the TAP-IVS mutation (T102I, A103V, and P106S) is a naturally occurring triple amino acid substitution linked to strong herbicide tolerance, and *OsSWEET11a* is a sugar transporter and a susceptibility gene hijacked by *Xoo* upon infection (Yang et al., 2006; Jiang et al., 2022). *Xoo* induces the expression of *OsSWEET11a* via the *pthXo1* TALE by binding to the EBE in the *OsSWEET11a* promoter region. Mutations in the EBE of *OsSWEET11a* have been shown to render rice resistant (Oliva et al., 2019). Constructs intended for both targets were generated using the modular assembly approach and were used for *Agrobacterium*-mediated rice transformation (Figure 4A and 4B). Twenty-one callus lines regenerated on medium supplemented with hygromycin, and each callus line produced multiple T_0_ plants. We considered plants that originated from a single callus line to be a single transformation event, and we performed mutation analysis on a mixture of all the T_0_ plants from each event. After confirming the presence of a transgene in all lines, we genotyped them for presence of the desired edits using the PCR-RE approach. The *BsrD*I restriction enzyme was used to detect edits in *OsEPSPS1*, as the TAP-IVS triple amino acid substitution leads to loss of the *BsrD*I site (Figure 4A). In *OsSWEET11a*, we intentionally incorporated a *Spe*I restriction sequence to disrupt the PthXo1 EBE and facilitate edit detection and genotyping (Figure 4B). As mentioned above, genotyping was performed on a mixture of plantlets that originated from a single callus line, and we considered sites with strong edited bands and weaker WT bands of the PCR amplicons on the agarose gel to be biallelic and sites with strong WT bands and weaker edited bands to be monoallelic. Sites with no band representing an edited site were considered to be WT sites (Figure 4C and Supplemental Figure 4A and 4B; Supplemental Table 4). Of the 21 lines, 16 were edited for either one or both genes, as detected by PCR-RE, reaching an editing frequency of 76.2% (Figure 4C and 4D and Supplemental Figure 4A and 4B; Supplemental Table 4). Of the 16 edited lines, 4 were edited for either *OsEPSPS1* or *OsSWEET11a*, and the remaining 12 were edited for both alleles. Co-editing efficiency of both target genes was thus 57.14% (Figure 4C and 4D and Supplemental Figure 4A and 4B; Supplemental Table 4).

To further validate the approach we used to classify edits as monoallelic or biallelic, we deep sequenced amplicons of both genes from different categories with various intensities of digestion to represent all the possibilities. Deep sequencing of *OsEPSPS1* revealed that some lines were purely monoallelic (#1 and #4), with >45% of reads mapping to the edited sequence, whereas one line was partially monoallelic (#5), with >25% of reads mapping to an edited allele (Supplemental Figure 5A). The same line (#5) was mainly edited (>50% reads) in another allele, and only the third amino acid of the TAP-IVS edit was changed (P > S) (Supplemental Figure 5A). For the biallelic lines (#13 and #14), >70% reads mapped to the edited allele. These results confirmed that the callus lines were mixtures, or chimeras, in which some shoots carried biallelic edits and others were monoallelic or WT. Different shoots could carry different edits, as seen in #5, in which partial editing was observed. We Sanger sequenced two lines (#4 and #13) for *OsEPSPS1* (Supplemental Figure 5C), and Sanger sequencing also validated the presence of the desired 3-bp substitution. For *OsSWEET11a*, we deep sequenced the amplicons of several lines and found that all lines carried an undesired deletion ranging from 19 to >40 bp in addition to the desired edit (Supplemental Figure 5B). These deletions may have originated from the ngRNA, which was used to enhance PE efficiency. Thus, further optimization is needed to decrease the frequency of by-product editing. Nevertheless, the *OsSWEET11a* edits knocked out the EBE from the promoter completely, and we decided to go forward with phenotyping evaluation. First, we screened individual tillers from some monoallelic and biallelic lines to identify the mutant biallelic tillers for phenotype analysis. We sprayed WT lines and lines carrying TAP-IVS edits with 4.2 mM glyphosate. The edited lines survived until maturity and seed set, whereas the WT lines died 5 days after spraying (Figure 4E). Similarly, we inoculated WT lines and lines carrying the *OsSWEET11a* EBE knockout edit with *Xoo* strain PXO99 and the ME2 strain carrying the *pthXo1* TALE gene. The edited lines were completely resistant to both strains, whereas the WT plants were completely susceptible (Figure 4F and 4G). These results indicate the feasibility of multiplex PE for simultaneous editing of both base substitutions and deletions. The knockouts generated in this case are easier to screen owing to the incorporation of a restriction site with the help of PE. This is an advantage of PE compared with CRISPR–Cas9 for knockout generation. However, further efforts are still needed to reduce the number of by-products generated during editing.

### Quadruplex prime editing efficiently edits four genes in the T_0_ generation

**Figure 5:**
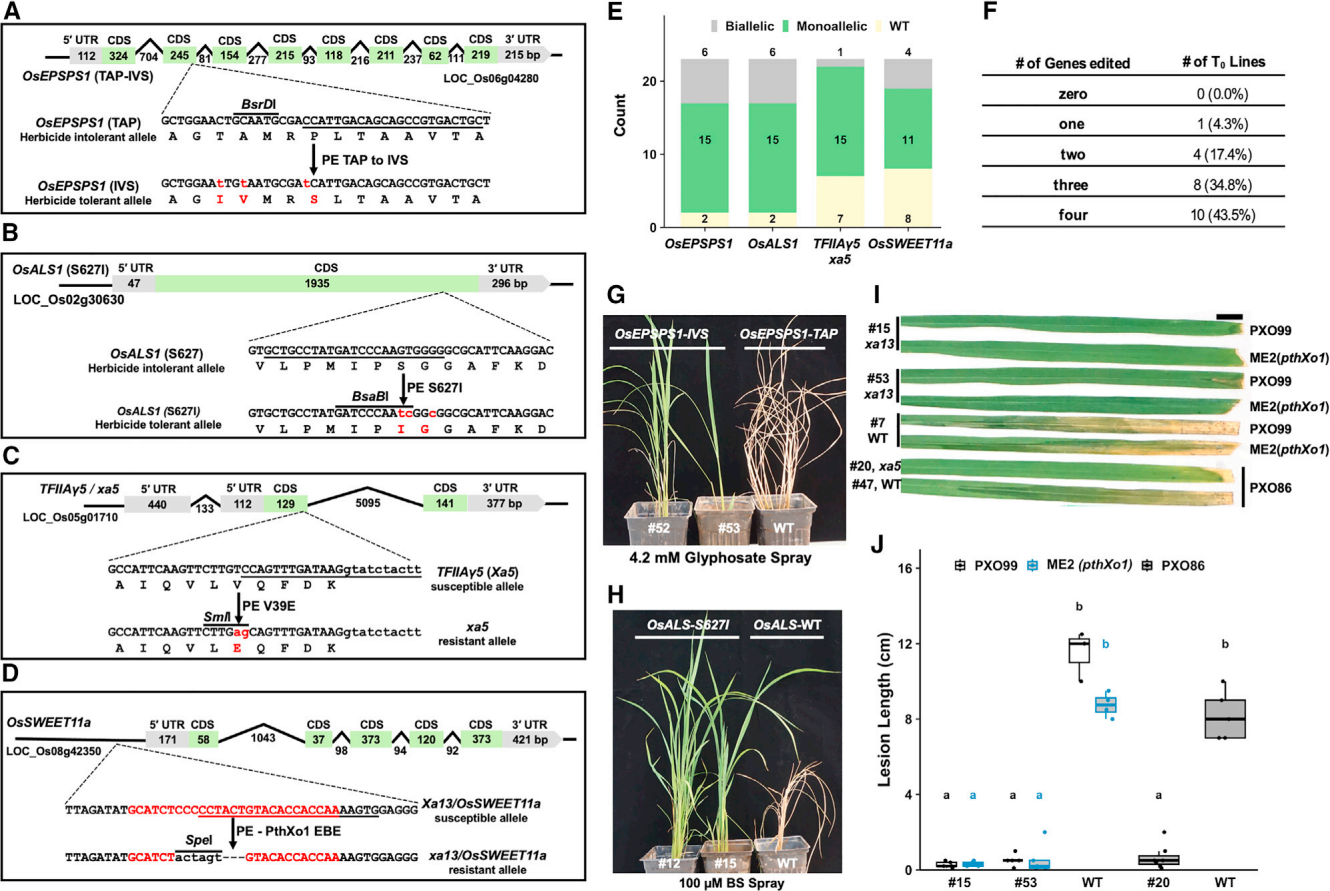
Quadruplex prime editing of *OsEPSPS1*, *OsALS1*, *TFIIAγ5*, and *OsSWEET11a* genes. **(A)** Gene structures of the *OsEPSPS1*-TAP allele and the *OsEPSPS1*-IVS allele. The PE target site (underlined) in the *OsEPSPS1*-TAP allele and the desired amino acid change (in red) in the *OsEPSPS1*-IVS allele are shown. **(B)** Structure of the OsALS1 gene with herbicide intolerant and tolerant alleles. The PE target site (underlined) in the *OsALS1-S627* allele and the desired amino acid change (in red) in the *OsALS1-I627* allele are shown. **(C)** Gene structures of *TFIIAγ5* and *xa5*. The PE target site (underlined) in the *TFIIAγ5* allele and the nick site (in red) in the edited strand of the *xa5* allele are shown. **(D)** Gene structures of *OsSWEET11a* and *xa13*. The effector binding element (EBE) in the promoter of susceptible *OsSWEET11a* is shown in red. The desired edit, including partial EBE deletion, and the *Spe*I recognition site insertion are shown in the resistant allele *xa13*. **(E)** Counts of monoallelic, biallelic, and WT lines based on PCR-RE of the T_0_ lines. **(F)** Summary of genotypes based on PCR-RE. **(G)** Treatment of *OsEPSPS1-*edited and WT lines with 4.2 mM glyphosate spray. Genotypes are shown at the top. Picture was taken 10 days after spraying. **(H)** Treatment of *OsALS1-*edited and WT lines with 100 μM bispyribac sodium (BS) spray. Genotypes are shown at the top. Picture was taken 10 days after spraying. **(I and J)** Disease phenotypes of edited biallelic lines. Lesion lengths were measured 12 days post inoculation with *Xoo* strains PXO99 and ME2(*pthXo1*) for *OsSWEET11a/xa13*-edited lines and PXO86 for *TFIIAγ5*-edited lines on three to five leaves of individual plants (n = 3–5). Scale bar, 1 cm. Letters a and b in **(J)** represent statistically significant differences in lesion lengths of edited versus WT lines for both PXO99 and ME2(*pthXo1*) for *OsSWEET11a/xa13*-edited lines and PXO86 for *TFIIAγ5*-edited lines calculated by Tukey’s test.

Next, we tested the editing efficiency of four targets using QPE. We selected two genes related to herbicide tolerance (*OsEPSPS1* and *OsALS1*) and two genes associated with bacterial blight resistance (*TFIIAγ5* and *OsSWEET11a*) (Figure 5A–5D). The targets and edits for *OsEPSPS1*, *TFIIAγ5*, and *OsSWEET11a* were the same as those in the DPE constructs. In *OsALS1*, a single amino acid substitution (S627I) has been shown to confer moderate bispyribac sodium tolerance (Li et al., 2022a). We generated constructs to edit these genes using modular assembly and transformed Kitaake using *Agrobacterium*. Twenty-three callus events regenerated on medium supplemented with hygromycin, and each callus line produced multiple T_0_ plants. As in the previous section, plants that originated from a single callus line were considered to represent single transformation events, and mutation analysis was performed on a mixture of all the T_0_ plants derived from individual callus lines. We first determined whether all the plants carried the nCas9 and the pegRNA units using PCR analysis (primer information in Supplemental Table 1). All plants were found to carry nCas9 and all four pegRNA units, ensuring stable transformation of multiple repeat units into the rice genome. We then genotyped the plants using the PCR-RE approach. *OsEPSPS1* had a loss of the *BsrD*I RE site due to the editing, *TFIIAγ5* gained an *Sml*I RE site with the edit, and *OsALS1* had a gain of *BsaB*I due to editing (Figure 5A–5C). The intentionally incorporated *Spe*I was used to genotype the *OsSWEET11a* edits (Figure 5D). We categorized monoallelic, biallelic, and WT lines using the approach described in the last section. PCR-RE on all four genes revealed that at least one site was edited in all 23 lines, making the editing efficiency 100% (Figure 5E and 5F and Supplemental Figure 6; Supplemental Table 5). One line had one site edited, five had two sites edited, eight had three sites edited, and ten lines had all four sites edited (Figure 5E and 5F and Supplemental Figure 6; Supplemental Table 5). Again, to validate this categorization approach and to accurately genotype the edited lines, we deep sequenced several lines for all four genes. For *OsEPSPS1*, at least 35% of reads mapped to the edited allele in the monoallelic lines (#7, #12, and #37), and at least 70% of reads mapped to the edited allele in the biallelic lines (#21, #28, #53, and #56) (Supplemental Figure 7A). Two lines (#12 and #56) also carried the partially edited allele in which only one amino acid (P > S) of three (TAP > IVS) was edited (Supplemental Figure 7A). For *OsALS1*, >55% of reads mapped to the edited allele in the monoallelic lines (#22, #37, and #59), and >85% of reads mapped to the edited allele in the biallelic lines (#7, #12, #15, #28, and #58) (Supplemental Figure 7B). In *TFIIAγ5*, >30% of reads mapped to the edited alleles in the monoallelic lines (#7, #15, #21, #28, and #50), and >75% of reads mapped to the edited allele in the single biallelic line (#20) (Supplemental Figure 7C). In *OsSWEET11a*, we observed by-product deletions ranging from 12 to more than 40 bp in addition to the desired edits. Some lines (#1 and #59) carried two alleles; one allele was perfectly edited, whereas the other contained the by-product deletion along with the desired edit (Supplemental Figure 7D). We further validated these results using Sanger sequencing of the *OsEPSPS1*, *OsALS1*, and *TFIIAγ5* genes (Supplemental Figure 8A–8C). All three genes were found to carry the desired edits in Sanger sequencing as well. Sanger sequencing of *OsSWEET11a* was attempted several times, but a good-quality read was never obtained; therefore, only deep-sequencing data for *OsSWEET11a* are presented here. Because the deletion in *OsSWEET11a* was only in the promoter region and did not reach to the coding region, and the other genes had desired edits only, we performed phenotype analysis of the edited lines under respective stresses (Figure 5G–5J). The lines edited for *OsEPSPS1* and *OsALS1* were sprayed with 4.2 mM glyphosate and 100 μM bispyribac sodium, respectively, and WT lines were sprayed as controls (Figure 5G and 5H). With the glyphosate spray, WT lines began to wilt as soon as 2 days post spray, and the edited lines remained unchanged. After 5 days, the WT lines had wilted completely, but the edited lines remained green, demonstrating that the *OsEPSPS1* edits were active in the T_0_ generation (Figure 5G). The effect of bispyribac sodium spray took longer: WT plants began to show wilting 6 days after spraying and had completely wilted 10 days after spraying. By contrast, the edited plants showed no obvious wilting 10 days after spraying, demonstrating that the *ALS1*-S627I edit was also active in the T_0_ lines (Figure 5H). However, the *OsALS1* mutant plants did not perform well and remain stunted compared with plants that did not receive any herbicide spray. This might be due to the moderate tolerance against bispyribac conferred by the single amino acid substitution, as opposed to the complete tolerance provided by the W548L and S627I mutations. Lines carrying the *OsSWEET11a* edits were resistant to *Xoo* strains PXO99 and ME2(*pthXo1*), and the line carrying the *xa5* edit was resistant to PXO86 (Figure 5I and 5J).

To further validate the multiplex PE system, we generated two additional constructs targeting three genes (TPE) and three additional constructs targeting four genes (QPE). In the first TPE construct, we targeted the genes *OsEPSPS1* for TAP-IVS mutation, *TFIIAγ5* for *xa5* (V39E) mutation, and *OsSPL14* (*SQUAMOSA promoter-binding protein like 14*) (Miura et al., 2010) for *IPA1* (Ideal plant architecture 1) (Jiao et al., 2010) mutation. We achieved an overall editing efficiency of 70.5% with this construct; 12 out of the 17 T_0_ lines were edited for at least one gene (Supplemental Table 6), with four edited for one gene, 7 edited for two genes, and 1 edited for all three genes (Supplemental Tables 6 and 7). Sanger sequencing confirmed that line #5 carried the desired edits in all three genes (Supplemental Figure 9A–9C). For the second TPE construct, we targeted *OsGS2* (*Grain size 2*) (Hu et al., 2015) to disrupt the microRNA 396 binding site while keeping the same amino acid sequence, along with *TFIIAγ5* for *xa5* (V39E) mutation and *OsSPL14* for *IPA1* mutation. The *OsGS2* pegRNA did not lead to any editing (validated with PCR-RE, Sanger sequencing, and deep sequencing). Of the total 25 lines, 8 were edited for *TFIIAγ5* to *xa5*, and 10 were edited for *OsSPL14* to *IPA1*; of these edited lines, 6 were edited for both *TFIIAγ5* to *xa5* and *OsSPL14* to *IPA1*. No line with edits in all three genes was obtained (Supplemental Tables 8 and 9).

For the first QPE construct, we targeted *OsGS2* to disrupt the microRNA 396 binding site, *OsSPL13* (*SQUAMOSA-promoter binding protein like 13*) for *GLW7* (*Grain length and weight 7*) (Si et al., 2016; Gupta et al., 2023a) mutation (originally the *GLW7* allele had a 6-bp deletion in the promoter; we replaced the 6 nt with the recognition site for *Spe*I), *TFIIAγ5* for *xa5* (V39E) mutation, and *OsSPL14* for *IPA1* mutation. The pegRNAs targeting genes *OsGS2* and *OsSPL13* did not lead to any editing in any of the T_0_ lines, as confirmed via PCR-RE, Sanger sequencing, and deep sequencing. The other two genes were edited at rates of 52.2% for *TFIIAγ5/xa5* and 47.8% for *OsSPL14/IPA1*, and the co-editing rate of the two genes was 21.7% of all lines (Supplemental Tables 10 and 11). Note that the pegRNA–ngRNA used for *OsGS2* was the same as that used in the previous TPE segment. We believe that this pegRNA–ngRNA has no activity for *OsGS2* editing, similar to *OsSPL13* editing. We decided to change the pegRNA–ngRNA of these two genes but, because of PAM (protospacer adjacent motif) restriction, we could change only the ngRNA. A new construct targeting the same four genes (*OsGS2*, *OsSPL13*, *TFIIAγ5*, and *OsSPL14*) but with a different ngRNA for *OsGS2* and *OsSPL13* was used for transformation of Kitaake. For *OsSPL13*, the new ngRNA led to 30% editing frequency based on PCR-RE, but no edits for *OsGS2* were recovered (Supplemental Tables 12 and 13). This suggests that the pegRNA of *OsGS2* has little to no activity. Overall, of the 20 T_0_ lines, 8 were edited for *OsSPL13*, *TFIIAγ5*, or *OsSPL14*; 6 were edited for two genes; and 1 was edited for three genes (Supplemental Tables 12 and 13). The *OsSPL13* edit was validated using Sanger sequencing (Supplemental Figure 10A). Finally, we replaced the *OsGS2* target with the *OsEPSPS1* target for another QPE experiment, thus targeting *OsEPSPS1*, *OsSPL13-2*, *TFIIAγ5*, and *OsSPL14* in one construct. Of the 18 T_0_ lines, 3 were unedited, 9 were edited for only one gene, 4 were edited for two genes, 2 were edited for three genes, and none were edited for all four genes (Supplemental Tables 14 and 15). The highest editing frequency was achieved for *OsEPSPS1*, with 13 lines carrying edits, followed by *TFIIAγ5*, with 4 lines edited, and *OsSPL13* and *OsSPL14*, each with 3 lines edited (Supplemental Tables 14 and 15).

## Discussion

The long-sought goal of biologists and crop breeders is to be able to precisely target and modify genes or genomes in living organisms. PE technology represents a significant advance toward achieving this capability (Jin et al., 2023). Over the past 3 years, remarkable progress has been made in enhancing PE efficiency in plants, elevating it from below 5% to nearly 100% (Jiang et al., 2020; Lin et al., 2020, 2021; Xu et al., 2020, 2021; Molla et al., 2021; Wang et al., 2021; Li et al., 2022a, 2022b; Zong et al., 2022; Gupta et al., 2023b; Jin et al., 2023; Ni et al., 2023; Qiao et al., 2023). This advance has now paved the way for efficient multigene targeting, a breakthrough that we demonstrate in this study. Although the prospect of multiplex PE has been demonstrated in wheat for up to eight genes (Ni et al., 2023) and in rice for up to three genes (Li et al., 2022a), the cloning of PE reagents has remained a daunting task, limiting its use to some labs with expertise in molecular cloning. By developing a modular assembly-based PE system for plants, we successfully targeted up to four genes in a single generation (Figure 1A–1K). The tandem pegRNA–ngRNA cassettes, although rich in repeats, remained stable in both *Escherichia coli* and *Agrobacterium*. In addition, the transgenes contained all four units (in the case of QPE) in multiple transformation events. Editing efficiency was found to be dependent on the activity of the pegRNA–ngRNA, and we did not observe any differences in editing rates based on the position of the pegRNA–ngRNA unit in the QPE. All units (except for the *OsGS2* pegRNA–ngRNA unit) were found to be active, resulting in mutations in the T_0_ generation. The introduction of modular assembly not only streamlines the cloning of PE-required reagents for single-gene targeting but also facilitates the targeting of multiple genes, thereby empowering numerous labs to leverage the full potential of PE for their genome-editing experiments. Furthermore, the system can easily be expanded to an even higher number of multiplexed pegRNA–ngRNA units.

In this study, we demonstrated the development and use of a multiplex PE system in rice to target traits related to disease resistance, herbicide tolerance, plant architecture, and grain yield, thereby harnessing the potential of PE to improve multiple agronomic traits in a single editing experiment. A bacterial disease of rice caused by *Xanthomonas oryzae* is the major threat to global rice production, and it can cause up to 70% yield loss in years of severe infection (Srinivasan and Gnanamanickam, 2005). In our previous study, we successfully employed PE to develop two distinct strategies to impart genetic resistance against bacterial blight of rice. The first strategy involved introduction of the EBE from the Os*SWEET14* gene into the promoter of the dysfunctional “Executor” *R* gene *xa23*, making it a functional *R* gene, *Xa23*^*SW14*^, and leading to dominant resistance that effectively protects rice against all *Xoo* strains carrying *pthXo3/*a*vrXa7* TALE genes. The second strategy relied on *xa5*, which conferred recessive resistance, offering protection against all Asian *Xoo* strains except those carrying the *pthXo1* TALE gene (Gupta et al., 2023b). To build upon these promising outcomes, we further employed DPE to combine the *Xa23*^*SW11*^ (in this case, the EBE from *OsSWEET11a* corresponding to the *pthXo1* TALE gene was incorporated into the promoter of *xa23*) and *xa5* edits in rice (Figures 2A–2G, 3A, and 3B). By creating this novel allelic combination not found in nature, we achieved robust and broad-spectrum resistance against all tested *Xoo* strains, including PXO99, which harbors the challenging *pthXo1* TALE gene (Figures 2A–2G, 3A, and 3B).

We incorporated a third strategy to provide genetic resistance against Asian *Xoo* strains by combining promoter EBE deletion/knockout of *OsSWEET11a* with the *xa5* edit. In the same construct, we edited two herbicide-related genes, *OsEPSPS1* and *OsALS1*, to their herbicide-tolerant alleles. In this QPE experiment, we achieved a high editing efficiency of 100%; all the lines were edited for at least one gene, and the co-editing efficiency for all four genes was 43.5%. We were able to detect completely biallelic or near biallelic edits (from a mixture of T_0_ lines originating from a single callus event) for all four genes in the T_0_ generation. Except for the *OsSWEET11a* EBE deletion, all genes had the desired edits, whereas *OsSWEET11a* had undesired deletions along with the desired edits (Figure 5A–5F and Supplemental Figures 7A–7D and 8A–8C; Supplemental Table 5). All edits were found to be active in the T_0_ generation as tested by challenging the edited plants with *Xoo* infection or herbicide spray (Figure 5G–5J). These results demonstrate the feasibility of multiplex PE for targeting multiple trait-related genes and testing the activity of new alleles in the T_0_ generation.

We validated the prospect of multiplex PE with five additional constructs that targeted either three or four genes concurrently. The editing efficiencies of these constructs varied depending upon the target and pegRNA–ngRNA used. Some targets were edited at very high rates, whereas others remained unedited or edited at lower rates. Because of the limitation of the PAM requirement, there is not much flexibility in terms of choosing a pegRNA, and PE rates are thus dependent on the activity of the pegRNA. The success of PE depends largely upon the activity of the pegRNA unit. In this study, we mainly selected pegRNAs that had previously shown activity in rice protoplasts or stable lines (Jiang et al., 2022; Gupta et al., 2023b), except for the pegRNAs of *OsSPL13*, *OsSPL14*, and *OsGS2.* This minimized the effort needed to optimize each pegRNA unit and ensured higher activity of these pegRNA units for testing the multiplexed system. Another component of the PE3 or PE5 system is the ngRNA, which nicks the unedited strand either upstream or downstream of the target region. Flexibility to choose the most active ngRNA near the target site requires optimization for every target. In our case, switching the ngRNA for *OsSPL13* targeting increased the editing rate from 0% to 30%, whereas changing the ngRNA for *OsGS2* had no effect, and no edits for *OsGS2* were obtained with any construct. Perhaps the *OsGS2* pegRNA had no or very low activity, and switching the ngRNA did not help in that case, whereas the *OsSPL13* pegRNA was active, and switching to an alternative ngRNA (perhaps with better activity than the first ngRNA) complemented the pegRNA to yield 30% editing in T_0_. This result highlights the need for further optimization of PE for recalcitrant targets and/or development of PAM-flexible or PAM-less Cas9 variants to be used for PE to allow selection of the best pegRNA for the target site.

Our results not only showcase the potential of multiplex PE in rice but also pave the way for more efficient and effective genetic resistance strategies against bacterial blight of rice. This strategy for design and construction of modular pegRNA–ngRNA units is readily applicable to multiplex PE in other crop species. The multiplex approach demonstrated in this study holds immense promise for significantly improving various agronomic traits simultaneously, providing a transformative and sustainable solution for rice production and food security.

## Methods

All primers used in this study are listed in Supplemental Table 1, and pegRNAs and ngRNAs are listed in Supplemental Table 2.

### Plant materials, bacterial strains, medium, and growth conditions

All editing experiments were performed using the *japonica* rice variety Kitaake (*Oryza sativa* spp. *japonica*). The *Xoo* strains used in the experiments were from the Yang laboratory’s collection. Rice plants were grown in a greenhouse and growth chambers with a 12-h/30°C light period and a 12-h/28°C dark period and a relative humidity of 60% to 75%. *E. coli* and *Agrobacterium tumefaciens* strains were cultivated in Luria-Bertani medium supplemented with appropriate antibiotics at temperatures of 37°C and 28°C, respectively. *Xoo* was grown on TSA (10 g/l tryptone, 10 g/l sucrose, 1 g/l glutamic acid, 1.5% Difco agar) at a temperature of 28°C. When necessary, the following concentrations of antibiotics were used: 25 μg/ml rifampicin, 50 μg/ml kanamycin, and 100 μg/ml spectinomycin.

### Disease assays

The leaf tip-clipping method was used to assess the disease phenotypes of edited rice as described previously (Yang and Bogdanove, 2013). In brief, *Xoo* glycerol stock stored at −80°C was streaked onto TSA (containing appropriate antibiotics) and grown at 28°C for approximately 3 days. Bacterial cells were then harvested from the plates, suspended in sterile water, washed twice, and resuspended in water. The optical density of the bacterial inoculum was adjusted to 0.5 at 600 nm. To perform the experiment, scissor blades were immersed in the *Xoo* suspension and used to clip the tips of fully expanded leaves. The resulting lesion lengths were measured either 12 days after inoculation or at specified time points. Each *Xoo* strain was tested with three to five replicates, each containing multiple leaves.

Data analysis was performed using R software, and the ggplot2 (Villanueva and Chen, 2019) and ggpubr (Kassambara and Kassambara, 2020) packages were used for plotting. The R package rstatix (Kassambara, 2020) was used to perform two-tailed Student’s *t*-tests, with or without Bonferroni correction for multiple comparisons. Tukey’s *post hoc* tests were performed using the R package agricolae (de Mendiburu and de Mendiburu, 2019).

### Development of the modular prime editing system

To develop the modular PE system, we digested the original PE3max vector with *Pme*I–*Afl*II to remove the 35S-CmYCLV-AtU6-pegRNA cassette and replaced it with *att*R1-ccdb-*att*R2 using a Gibson assembly kit (New England Biolabs), resulting in pG3H-PE3max-attR1R2. The resulting vector served as the destination vector for PE cloning. To construct the entry vectors, the 35S-CmYCLV-AtU6-pegRNA cassette was synthesized as gBlock from Integrated DNA Technologies and cloned into pCR8-attL1-attL2, pCR8-attL1-attR5, pCR8-attL5-attL2, pCR8-attL5-attL4, pCR8-attR4-attL2, pCR8-attR4-attR3, and pCR8-attL3-attL2 vectors between the *att* regions, resulting in modular pCR8-pegRNA-ngRNA entry vectors. The double-stranded oligonucleotides with proper 4-nt overhangs at each side for the pegRNA spacer were first cloned at the *Bsm*BI site, and, similarly, oligonucleotides corresponding to the extension RNA region and ngRNA were sequentially cloned at the *Bsa*I sites of the respective pCR8-pegRNA vectors. All plasmids were confirmed by whole-plasmid sequencing via Plasmidsaurus. A detailed protocol for the design of pegRNA–ngRNAs and their subsequent cloning entry vectors and destination vector is provided in the Supplemental Protocol.

### Rice transformation

Kitaake rice was transformed with the *Agrobacterium*-based DNA delivery method with slight modifications, following the procedure described by Hiei et al. (1994). In brief, mature seed embryos of Kitaake were used for callus induction in Murashige and Skoog (MS) medium supplemented with 2 mg/l 2,4-dichlorophenoxyacetic acid. Callus cells derived from the scutella were co-cultivated with *Agrobacterium* strain LBA4404/pVS1-VIR2 carrying the appropriate PE plasmids. The inoculated callus cells were cultured in MS medium supplemented with 2,4-dichlorophenoxyacetic acid (2 mg/l), hygromycin (50 mg/l), and Timentin (200 mg/l) for two rounds of selection (14 days per round) to generate hygromycin-resistant callus lines. The hygromycin-resistant callus lines were then transferred to a regeneration medium (MS supplemented with BAP and NAA) to induce formation of embryogenic shoots. The developed shoots were transferred to a rooting medium (½ MS medium supplemented with 25 mg/l hygromycin) to facilitate root formation, then transferred to soil and cultivated in a greenhouse.

### RNA isolation and gene expression analysis

RNA was extracted from the leaves of PE-edited and WT Kitaake lines that had been infiltrated with *Xoo* inoculum using a needleless syringe. DNase I treatment (Thermo Fisher Scientific) was applied to eliminate any remaining DNA. RNA quality was evaluated using agarose gel electrophoresis, and RNA concentration was measured using a NanoDrop instrument (Thermo Fisher Scientific). First-strand cDNA was synthesized from 1 μg of RNA using the iScript cDNA synthesis kit (Bio-Rad). The resulting cDNA was diluted at 1:20 for use in RT-PCR and RT-qPCR with gene-specific primers. For RT-qPCR, PowerTrack SYBR master mix (Thermo Fisher Scientific) was used. *OsActin* was used as the housekeeping control gene, and the 2^−ΔΔCt^ method was used to calculate the fold change.

### Genotyping of PE callus lines and T_0_ and T_1_ plants and deep sequencing analysis

DNA was isolated from T_0_ and T_1_ lines using the CTAB method. To detect editing events, primers flanking the target sites were used for PCR amplification of the specific regions, which were then digested with appropriate enzymes. The PCR amplicons from the edited lines were subjected to deep sequencing using the Illumina MiSeq instrument (PE150). In brief, the 150- to 250-bp region flanking the target site was first amplified in the initial PCR round using gene-specific primers extended with sequencing primers. Subsequently, a second nested PCR was performed using dual barcoded Illumina adapters to amplify the gene-specific products from the first round. The resulting PCR products were purified using columns, pooled in equal quantities, and sent for sequencing at the DNA sequencing core facility of the University of Missouri–Columbia and to Azenta–GENEWIZ for deep sequencing. The obtained reads were demultiplexed and trimmed during the sequencing process. For analysis, CRISPResso2 was used with default settings for both NHEJ and PE output (Pinello et al., 2016).

### Herbicide treatment

*EPSPS1*-edited plants were sprayed with 2 ml/l (4.2 mM) commercial glyphosate (Monsanto), and pictures were taken 10 days after treatment. *ALS1*-edited plants were sprayed with 100 μM bispyribac sodium salt, and pictures were taken 10 days post spraying.

### Statistics and data analysis

Data were analyzed and plotted using the R packages ggplot2 (Villanueva and Chen, 2019), ggpubr (Kassambara and Kassambara, 2020), rstatix (Kassambara, 2020), and agricolae (de Mendiburu and de Mendiburu, 2019). Tukey’s test was used for all figures with statistics.

## Data and code availability

The plant materials and constructs generated in this study are available upon request.

## Funding

The work was partially supported by an NSF award (IOS-2210259 to B.Y.) and a subaward to the University of Missouri from the Heinrich Heine University of Dusseldorf funded by the 10.13039/100000865Bill & Melinda Gates Foundation (OPP1155704). A.G. is partially supported by the Daniel Millikan Award for Outstanding Research in Plant–Microbe Interactions at the University of Missouri.

## Author contributions

A.G. and B.Y. designed the research; A.G., B.L., and S.R. performed the research; Q.-J.C., A.G., and B.Y. analyzed the data; and A.G. and B.Y. wrote the paper with revisions from the other authors.

## Supplemental information

Document S1. Supplemental Figures 1–10, Supplemental Tables 1–15, Supplemental Sequences 1–12, and Supplemental Protocol

Document S2. Article plus supplemental information
