# Supplementary material for: Modularly assembled multiplex prime editors for simultaneous editing of agronomically important genes in rice
Source: Plant Commun. 2023 Oct 26;5(2):100741. doi: 10.1016/j.xplc.2023.100741 (PMC10873889; doi:10.1016/j.xplc.2023.100741)
Supplement: Document S1. Supplemental Figures 1–10, Supplemental Tables 1–15, Supplemental Sequences 1–12, and Supplemental Protocol [file mmc1.pdf]

Plant Communications, Volume *5*

Supplemental information

Modularly assembled multiplex prime editors for simultaneous editing of agronomically important genes in rice

Ajay Gupta, Bo Liu, Saad Raza, Qi-Jun Chen, and Bing Yang

Modularly assembled multiplex prime editors for simultaneous editing of agronomically important genes in rice

Ajay Gupta[1], Bo Liu[1], Saad Raza[1], Qi-Jun Chen[2,3], Bing Yang[1,4, *]

[1] Division of Plant Science and Technology, Bond Life Sciences Center, University of Missouri, Columbia, MO 65211, USA
[2] State Key Laboratory of Plant Physiology and Biochemistry, College of Biological Sciences, China Agricultural University, Beijing 100193, China
[3] Center for Crop Functional Genomics and Molecular Breeding, China Agricultural University, Beijing 100193, China
[4] Donald Danforth Plant Science Center, St. Louis, MO 63132, USA

* Corresponding author
Bing Yang (yangbi@missouri.edu)

Supplemental Information:

Supplemental Figure 1 - 10
Supplemental Table 1- 15
Supplemental Sequence 1 – 12
Supplemental Protocol

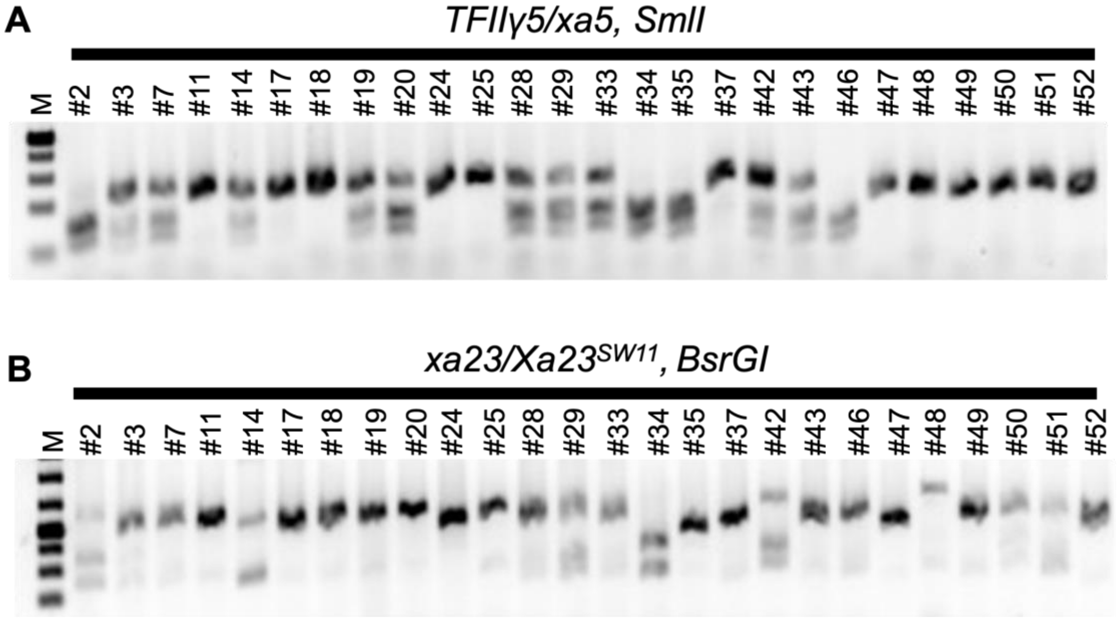

Supplemental Figure 1. (A) Genotyping of *TFIIAγ5/xa5* edited lines as indicated above lanes with *Sml*I digestion of relevant PCR-amplicons. Editing introduced a *Sml*I site and led to two smaller bands (biallelic edits, e.g., #2), three bands (monoallelic edits, e.g., #3) or one band for wild type genotype (e.g., #11) after treatment of PCR amplicons with *Sml*I. (B) Genotyping of *xa23*/*Xa23*^{SW11}edited lines as indicated above lanes with *Bsr*GI digestion of relevant PCR-amplicons. Editing introduced a *Bsr*GI site and led to two smaller bands (biallelic edits, e.g., #34), three bands (monoallelic edits, e.g., #29) or one band for wild type (e.g., #11) after treatment of PCR amplicons with *BsrG*I.

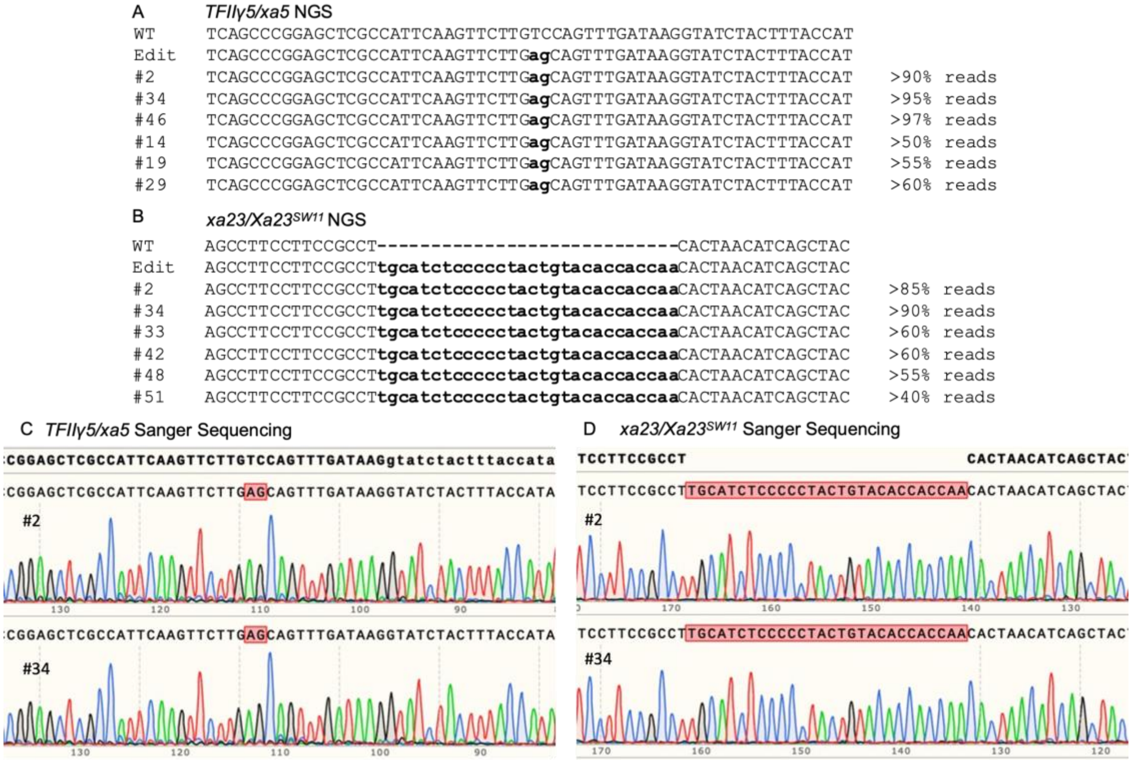

Supplemental Figure 2. (A) Next generation deep-sequencing of *TFIIAγ5/xa5* edited lines. (B) Next generation deep-sequencing of *xa23*/*Xa23*^{SW11} edited lines. Wildtype (WT) and edited sequences are shown in the first and second lane, respectively. Edited nucleotides are in bold. T_0 line number is indicated on the right and the percent of reads belonging to the edited is indicated on the right. Sanger sequencing the successful editing to *xa5* (C) and *Xa23*^{SWT11} (D) alleles. Letters shaded in red are edited nucleotides.

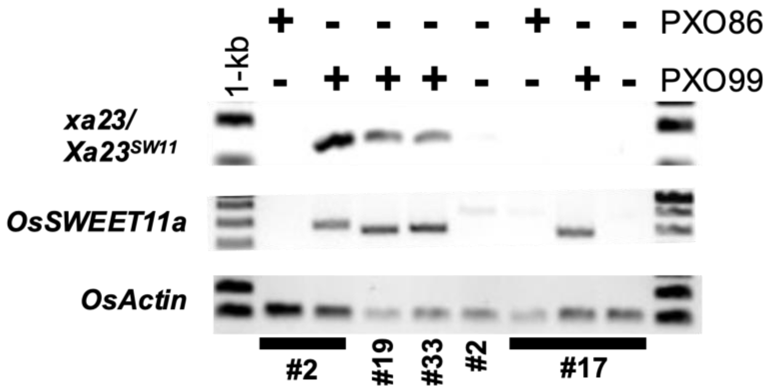

Supplemental Figure 3. RT-PCR of *xa23/Xa23*^{SW11} and *OsSWEET11a* gene. *OsActin* (housekeeping control) gene amplified in all lines is used as a control. Infiltration with either PXO99 or PXO86 is indicated with + and – on the top of gel lanes. Number of T_0 lines infiltrated is indicated at the bottom of the gel images.

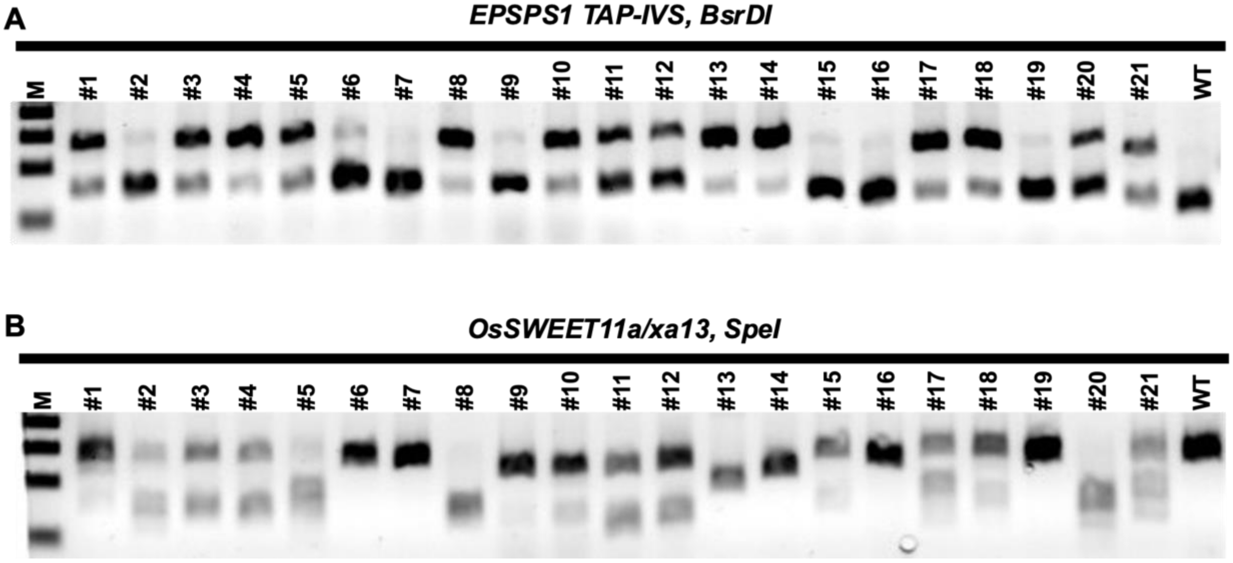

Supplemental Figure 4. (A) Genotyping of *OsEPSPS1* edited lines as indicated above lanes with *BsrD*I digestion of relevant PCR-amplicons. Editing led to elimination of the *BsrD*I restriction site relative to the wild type allele. (B) Genotyping of *OsSWEET11a/xa13* edited lines as indicated above lanes with *Spe*I digestion of relevant PCR-amplicons. Editing resulted in presence of *Spe*I restriction sequence compared to the wild type allele.

A OsEPSPS1 NGS

WT	ACTCTTCTTGGGGAACGCTGGAACTGCAATGCGACCATTGACAGCAGCCGTGACTGCTGC	
Edit	ACTCTTCTTGGGGAACGCTGGAA**t**TG**t**AATGCGA**t**CATTGACAGCAGCCGTGACTGCTGC	
#1	ACTCTTCTTGGGGAACGCTGGAA**t**TG**t**AATGCGA**t**CATTGACAGCAGCCGTGACTGCTGC	>45% reads
#3	ACTCTTCTTGGGGAACGCTGGAA**t**TG**t**AATGCGA**t**CATTGACAGCAGCCGTGACTGCTGC	>95% reads
#4	ACTCTTCTTGGGGAACGCTGGAA**t**TG**t**AATGCGA**t**CATTGACAGCAGCCGTGACTGCTGC	>65% reads
#5	ACTCTTCTTGGGGAACGCTGGAA**t**TG**t**AATGCGA**t**CATTGACAGCAGCCGTGACTGCTGC	>25% reads
#5	ACTCTTCTTGGGGAACGCTGGAACTGCAATGCGA**t**CATTGACAGCAGCCGTGACTGCTGC	>50% reads
#13	ACTCTTCTTGGGGAACGCTGGAA**t**TG**t**AATGCGA**t**CATTGACAGCAGCCGTGACTGCTGC	>70% reads
#14	ACTCTTCTTGGGGAACGCTGGAA**t**TG**t**AATGCGA**t**CATTGACAGCAGCCGTGACTGCTGC	>75% reads

B *OsSWEET11a* NGS

WT	TGCATCTCCCCCTACTGTACACCACCAAAAGTGGAGGGTCTCCAACTATATAAACACTGAGCC	
Edit	TGCATCT**A**C---TA**G**TGTACACCACCAAAAGTGGAGGGTCTCCAACTATATAAACACTGAGCC	
#2	TGCATCT**A**C---TA**G**TGTACAC-----------------------------------TGAGCC	>90% reads
#3	TGCATCT**A**C---TA**G**TGTACACCACCA-------------------TATATAAACACTGAGCC	>75% reads
#4	TGCATCT**A**C---TA**G**TGTACACCAC---------------------TATATAAACACTGAGCC	>75% reads
#5	TGCATCT**A**C---TA**G**TGTACACCAC--------------------------------------	>50% reads
#8	TGCATCT**A**C---TA**G**-----------------------TCTCCAACTATATAAACACTGAGCC	>95% reads
#11	TGCATCT**A**C---TA**G**TGTACACCA**TA**A**T**A**T**G**C**-------------CTAT**TCTT**A**G**A**GA**GAG**GT**	>65% reads

C OsEPSPS1 Sanger Sequencing

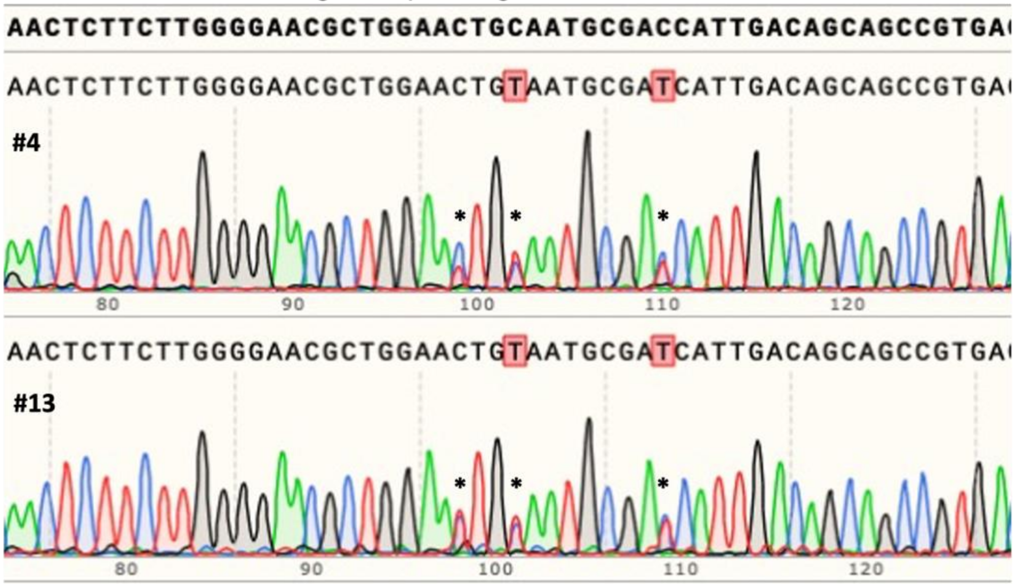

Supplemental Figure 5. (A) Next generation deep-sequencing of *OsEPSPS1* edited lines. (B) Next generation deep-sequencing of *OsSWEET11a* edited lines. Wildtype (WT) and edited sequence is shown in the first and second lane respectively. Edited nucleotides are in bold. T_0 line number is indicated on the right and the percent of reads belonging to the edited is indicated on the right. Sanger sequencing the successful editing of *OsEPSPS1* (C) and *OSWEET11a* (D) alleles. Letters shaded in red are edited nucleotides.

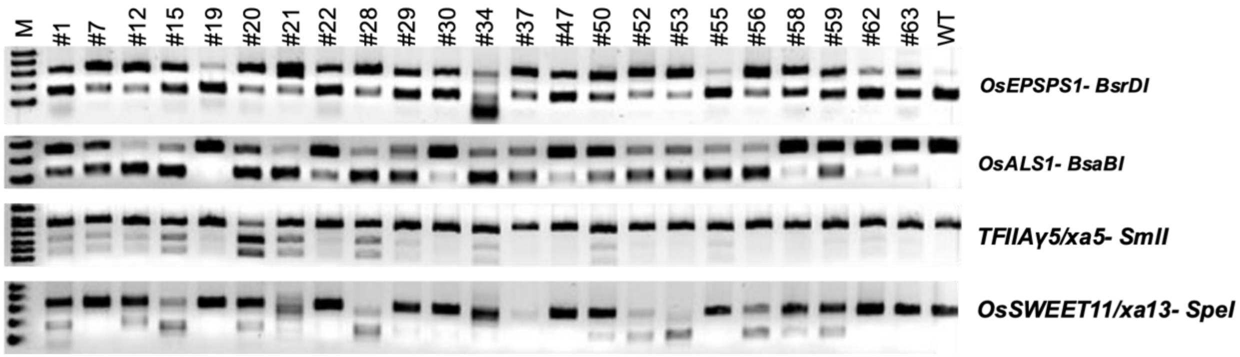

Supplemental Figure 6. Genotyping of the edited rice lines using PCR-RE approach. The gene names for amplicons and restriction enzymes used are indicated on the side of the gel images. Line numbers are denoted on the top of the gels. Editing resulted in loss of *BsrD*I site in *OsEPSPS1*, and gain of *BsaB*I site, *Sml*I site, and *Spe*I site in *OsALS1*, *TFIIAγ5/xa5*, and *OsSWEET11a* respectively.

A *OsEPSPS1* NGS

WT	ACTCTTCTTGGGGAACGCTGGAACTGCAATGCGACCATTGACAGCAGCCGTGACTGCTGC	
Edit	ACTCTTCTTGGGGAACGCTGGAA**t**TG**t**AATGCGA**t**CATTGACAGCAGCCGTGACTGCTGC	
#7	ACTCTTCTTGGGGAACGCTGGAA**t**TG**t**AATGCGA**t**CATTGACAGCAGCCGTGACTGCTGC	>45% reads
#12	ACTCTTCTTGGGGAACGCTGGAA**t**TG**t**AATGCGA**t**CATTGACAGCAGCCGTGACTGCTGC	>55% reads
#12	ACTCTTCTTGGGGAACGCTGGAACTGCAATGCGA**t**CATTGACAGCAGCCGTGACTGCTGC	>40% reads
#21	ACTCTTCTTGGGGAACGCTGGAA**t**TG**t**AATGCGA**t**CATTGACAGCAGCCGTGACTGCTGC	>85% reads
#28	ACTCTTCTTGGGGAACGCTGGAA**t**TG**t**AATGCGA**t**CATTGACAGCAGCCGTGACTGCTGC	>75% reads
#37	ACTCTTCTTGGGGAACGCTGGAA**t**TG**t**AATGCGA**t**CATTGACAGCAGCCGTGACTGCTGC	>35% reads
#52	ACTCTTCTTGGGGAACGCTGGAA**t**TG**t**AATGCGA**t**CATTGACAGCAGCCGTGACTGCTGC	>95% reads
#53	ACTCTTCTTGGGGAACGCTGGAA**t**TG**t**AATGCGA**t**CATTGACAGCAGCCGTGACTGCTGC	>70% reads
#56	ACTCTTCTTGGGGAACGCTGGAA**t**TG**t**AATGCGA**t**CATTGACAGCAGCCGTGACTGCTGC	>70% reads
#56	ACTCTTCTTGGGGAACGCTGGAACTGCAATGCGA**t**CATTGACAGCAGCCGTGACTGCTGC	>20% reads

B *OsALS1* NGS

WT	CACCAGGAGCATGTGCTGCCTATGATCCCAAGTGGGGGCGCATTCAAGGACATGATCCTG	
Edit	CACCAGGAGCATGTGCTGCCTATGATCCCAA**tc**GG**c**GGCGCATTCAAGGACATGATCCTG	
#7	CACCAGGAGCATGTGCTGCCTATGATCCCAA**tc**GG**c**GGCGCATTCAAGGACATGATCCTG	>85% reads
#12	CACCAGGAGCATGTGCTGCCTATGATCCCAA**tc**GG**c**GGCGCATTCAAGGACATGATCCTG	>99% reads
#15	CACCAGGAGCATGTGCTGCCTATGATCCCAA**tc**GG**c**GGCGCATTCAAGGACATGATCCTG	>90% reads
#22	CACCAGGAGCATGTGCTGCCTATGATCCCAA**tc**GG**c**GGCGCATTCAAGGACATGATCCTG	>55% reads
#28	CACCAGGAGCATGTGCTGCCTATGATCCCAA**tc**GG**c**GGCGCATTCAAGGACATGATCCTG	>95% reads
#37	CACCAGGAGCATGTGCTGCCTATGATCCCAA**tc**GG**c**GGCGCATTCAAGGACATGATCCTG	>60% reads
#58	CACCAGGAGCATGTGCTGCCTATGATCCCAA**tc**GG**c**GGCGCATTCAAGGACATGATCCTG	>95% reads
#59	CACCAGGAGCATGTGCTGCCTATGATCCCAA**tc**GG**c**GGCGCATTCAAGGACATGATCCTG	>75% reads

C *TFIIγ5/xa5* NGS

WT	TCAGCCCGGAGCTCGCCATTCAAGTTCTTGTCCAGTTTGATAAGGTATCTACTTTACCAT	
Edit	TCAGCCCGGAGCTCGCCATTCAAGTTCTTG**ag**CAGTTTGATAAGGTATCTACTTTACCAT	
#7	TCAGCCCGGAGCTCGCCATTCAAGTTCTTG**ag**CAGTTTGATAAGGTATCTACTTTACCAT	>30% reads
#15	TCAGCCCGGAGCTCGCCATTCAAGTTCTTG**ag**CAGTTTGATAAGGTATCTACTTTACCAT	>40% reads
#20	TCAGCCCGGAGCTCGCCATTCAAGTTCTTG**ag**CAGTTTGATAAGGTATCTACTTTACCAT	>75% reads
#21	TCAGCCCGGAGCTCGCCATTCAAGTTCTTG**ag**CAGTTTGATAAGGTATCTACTTTACCAT	>50% reads
#28	TCAGCCCGGAGCTCGCCATTCAAGTTCTTG**ag**CAGTTTGATAAGGTATCTACTTTACCAT	>40% reads
#50	TCAGCCCGGAGCTCGCCATTCAAGTTCTTG**ag**CAGTTTGATAAGGTATCTACTTTACCAT	>35% reads

D *OsSWEET11a* NGS

WT	TGCATCTCCCCCTACTGTACACCACCAAAAGTGGAGGGTCTCCAACTATATAAACACTGA	
Edit	TGCATCT**A**C---TA**G**TGTACACCACCAAAAGTGGAGGGTCTCCAACTATATAAACACTGA	
#1	TGCATCT**A**C---TA**G**TGT**T**---------------------------TATATAAACACTGA	>50% reads
#1	TGCATCT**A**C---TA**G**TGTACACCACCAAAAGTGGAGGGTCTCCAACTATATAAACACTGA	>20% reads
#15	TGCATCT**A**C---TA**G**TGTACA------------------------------------TGA	>90% reads
#21	TGCATCT**A**C---TA**G**T**A**---GA	>90% reads
#52	TGCATCT**A**C---TA**G**TGTACACCACC----------------------------------	>90% reads
#53	TGCATCT**A**C---TA**G**TGTACAC-----------------------------------TGA	>90% reads
#59	TGCATCT**A**C---TA**G**TG**CTACT**------------AGGGTCTCCAACTATATAAACACTGA	>50% reads
#59	TGCATCT**A**C---TA**G**TGTACACCACCAAAAGTGGAGGGTCTCCAACTATATAAACACTGA	>10% reads

Supplemental Figure 7. Next generation deep-sequencing of *OsEPSPS1* edited lines (A), *OsALS1* edited lines (B), *TFIIγ5/xa5* edited lines (C), *OsSWEET11a* edited lines (D). Edited nucleotides are in bold. T_0 line number is indicated on the right and the percent of reads belonging to the edited is indicated on the right.

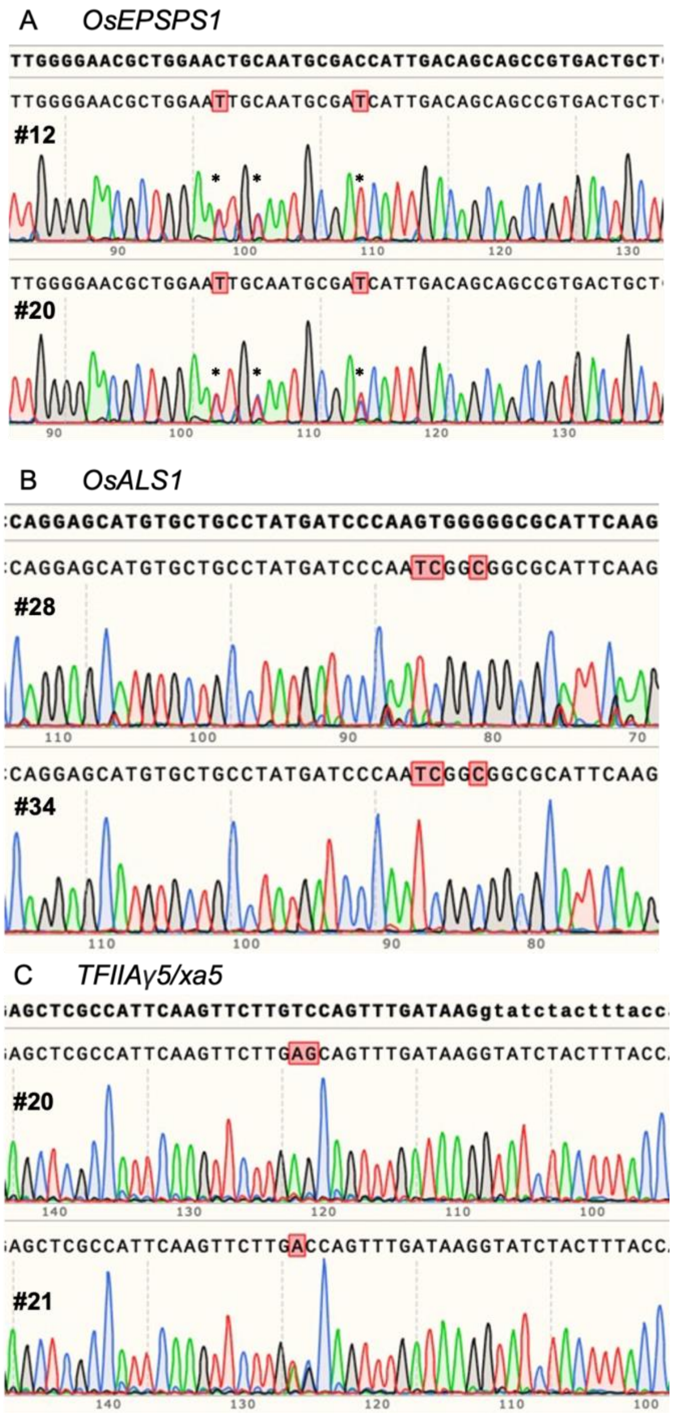

Supplemental Figure 8. Sanger sequencing of *OsEPSPS1* (A), *OsALS1* (B), and *xa5* (C) in quadruplex prime editing (QPE). Letters shaded in red (if present) are edited nucleotides. * Represents the 3 intended nucleotide substitutions in (A).

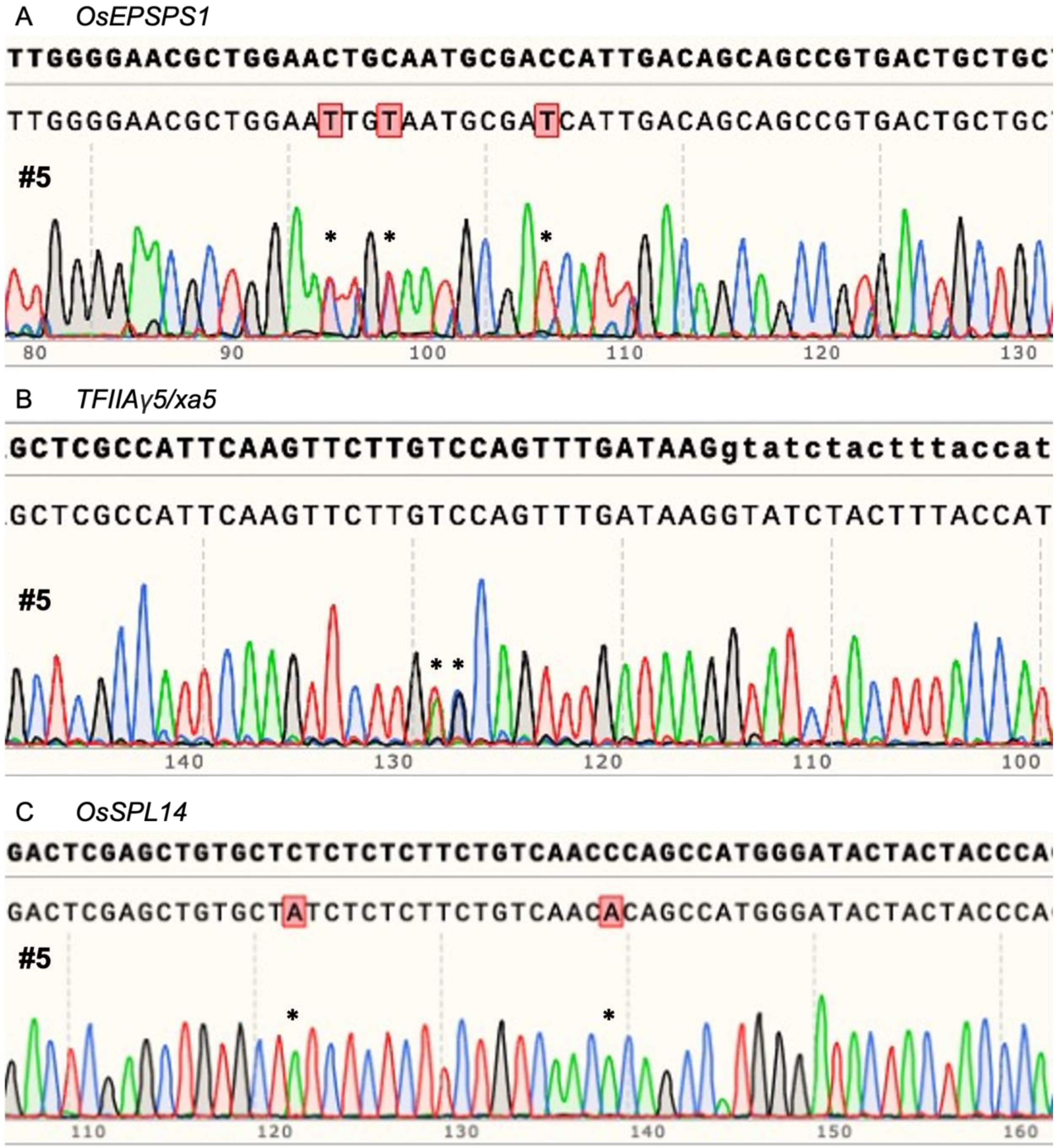

Supplemental Figure 9. Sequencing of *OsEPSPS1* (A), *xa5* (B), and *OsSPL14* (C) in triplex prime editing (TPE). Letters shaded in red (if present) are edited nucleotides. * Represent the position of intended nucleotide substitutions.

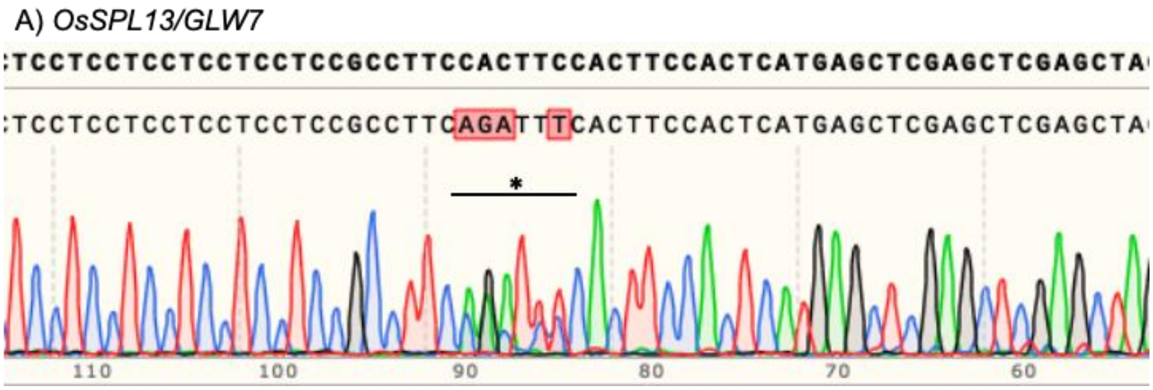

Supplemental Figure 10. Sequencing of *OsSPL13* in quadruple prime editing (QPE). Letters shaded in red are edited nucleotides. * Represent the position of intended nucleotide substitutions.

Supplemental Table 1. Sequence of oligonucleotides

Name	Sequence (5’ to 3’)	Purpose
attR1-F7	TCCTGTCAAACACTGATAGTTTAAAGCTAgagacGGGGATCACAAGTTTG	To construct pG3H-PE3max/PE5max-GW-ccdb-attL1-attL2
attR1-R7	CACGCTGCACTGCAGGCATGCAAGCTTCACCACTTTGTACAAGAAAGCTG	
pegXa5-F1	tgcaAAGTAGATACCTTATCAAAC	To make pegRNA/ngRNA in entry vectors to target *TFIIAγ5* and to screen for pegRNA-ngRNA in plants
pegXa5-R1	aaacGTTTGATAAGGTATCTACTT	
extXa5-F1	gtgcGCCATTCAAGTTCTTGagCAGTTTGATAAGGTATCAGGAATAA	
extXa5-R1	cgcgTTATTCCTGATACCTTATCAAACTGctCAAGAACTTGAATGGC	
ngXa5-F1	gataAGTTCTTGagCAGTTTGATA	
ngXa5-R1	aaacTATCAAACTGctCAAGAACT	
pegXa23-F1	tgcaGTAGCTGATGTTAGTGAGG	To make pegRNA/ngRNA in entry vectors to target *xa23* and to screen for pegRNA-ngRNA in plants
pegXa23-R1	aaacCCTCACTAACATCAGCTAC	
extXa23PthXo1-F1	gtgcGCCTTCCTTCCGCCTtgcatctcccctactgtacaccaccaaCACTAACATCAtaaggaga	
extXa23PthXo1-R1	cgcgtctccttaTGATGTTAGTGttggtggtgtacagtaggggagatgcaAGGCGGAAGGAAGGC	
ngXa23-F2	gataGTTCCTTGTTGCATCATCTCA	
ngXa23-R2	aaacTGAGATGATGCAACAAGGAAC	
pegOsEPSPS-F1	tgcaGCAGTCACGGCTGCTGTCAA	To make pegRNA/ngRNA in entry vectors to target *OsEPSPS1* and to screen for pegRNA-ngRNA in plants
pegOsEPSPS-R1	aaacTTGACAGCAGCCGTGACTGC	
extOsEPSPS-F1	gtgcTGGAAtTGtAATGCGAtCATTGACAGCAGCCGTGAAGAAATAT	
extOsEPSPS-R1	cgcgATATTTCTTCACGGCTGCTGTCAATGaTCGCATTaCAaTTCCA	
ngOsEPSPS-F1	gataTGTTGAGAAGGATGCGAAAG	
ngOsEPSPS-R1	aaacCTTTCGCATCCTTCTCAACA	
pegOsALS-F1	tgcaGCTGCCTATGATCCCAAGTG	To make pegRNA/ngRNA in entry vectors to target *OsALS1* and to screen for pegRNA-ngRNA in plants
pegOsALS-R1	aaacCACTTGGGATCATAGGCAGC	
extOsALS-F1	gtgcGCCgCCgaTTGGGATCATAGGCTTAAACAG	
extOsALS-R1	cgcgCTGTTTAAGCCTATGATCCCAAtcGGcGGC	

ngOsALS-F1<br>ngOsALS-R1	gataTCCTTGAATGCGCCgCCgaT<br>aaacAtcGGcGGCGCATTCAAGGA	
pegSWEET11-F1<br>pegSWEET11-R1<br>extSWEET11-F1<br>extSWEET11-R1<br>ngSWEET11-F1<br>ngSWEET11-R1	tgcaCACTTTTGGTGGTGTACAGT<br>aaacACTGTACACCACCAAAAGTG<br>gtgcAAAGGTTAGATATGCATCTactagtGTACACCACCAATTTATAAA<br>cgcgTTTATAAATTGGTGGTGTACactagtAGATGCATATCTAACCTTT<br>gataCtagtGTACACCACCAAAAG<br>aaacCTTTTGGTGGTGTACactag	To make pegRNA/ngRNA in entry vectors to target *OsSWEET11* and to screen for pegRNA-ngRNA in plants
pegGS2-F1<br>pegGS2-R1<br>extGS2-F1<br>extGS2-R1<br>ngGS2-F1<br>ngGS2-R1<br>ngGS2-F2<br>ngGS2-R2	tgcaGCGACCAGCTGCGTTTCCAC<br>aaacGTGGAAACGCAGCTGGTCGC<br>gtgcACCGCGGCCGCAACCGcagccGcAAGCCgGTcGAAACGCAGCACATACAG<br>gcgcCTGTATGTGCTGCGTTTCgACcGGCTTgCggctgCGGTTGCGGCCGCGGT<br>gataGCCGCAACCGcagccGcAAGC<br>aaacGCTTgCggctgCGGTTGCGGC<br>gataGAAATGGCGGTGCTCGAAGG<br>aaacCCTTCGAGCACCGCCATTTC	To make pegRNA/ngRNA in entry vectors to target *OsGS2* and to screen for pegRNA-ngRNA in plants
pegSPL13-F1<br>pegSPL13-R1<br>extSPL13-F1<br>extSPL13-R1<br>ngSPL13-F1<br>ngSPL13-R1<br>extSPL13-F2<br>extSPL13-R2<br>ngSPL13-F2<br>ngSPL13-R2	tgcaGCTCGAGCTCGAGCTCATGAG<br>aaacCTCATGAGCTCGAGCTCGAGC<br>gtgcTCCTCCTCCGCCTTCCACTTCCACTCATGAGCTCGAGCTAAATATAT<br>cgcgATATATTTAGCTCGAGCTCATGAGTGGAAGTGGAAGGCGGAGGAGGA<br>gataGCCACCGCTTCCACCTCCCCT<br>aaacAGGGGAGGTGGAAGCGGTGGC<br>gtgcCTTCagatctCACTTCCACTCATGAGCTCGAGCTattaactt<br>cgcgAagttaatAGCTCGAGCTCATGAGTGGAAGTGagatctGAAG<br>gataGCACCCTGCCTCCACACACA<br>aaacTGTGTGTGGAGGCAGGGTGC	To make pegRNA/ngRNA in entry vectors to target *OsSPL13* and to screen for pegRNA-ngRNA in plants

pegSPL14-F1<br>pegSPL14-R1<br>extSPL14-F1<br>extSPL14-R1<br>ngSPL14-F1<br>ngSPL14-R1	tgcaGGTAGTAGTATCCCATGGCT<br>aaacAGCCATGGGATACTACTACC<br>gtgcTGTGCTaTCTCTCTTCTGTCAACaCAGCCATGGGATACTAAGGAATAA<br>cgcgTTATTCCTTAGTATCCCATGGCTGtGTTGACAGAAGAGAGAtAGCACA<br>gataGCTGGCCCAAATCTCCCTCC<br>aaacGGAGGGAGATTTGGGCCAGC	To make pegRNA/ngRNA in entry vectors to target *OsSPL14* and to screen for pegRNA-ngRNA in plants
yCLV-F1<br>pegHSP-R1	TAGCCTAGAAGTAGTCAAGG<br>CAATTAGCCCCGGAGATATC	Screening and sequencing of PE entry vectors
ZmUbi-F<br>Cas9-R	CCCTGCCTTCATACGCTATT<br>AGCTTCTTCCGCAGATGGTA	To screen for Cas9 in plants
Ga5RT-F<br>Xa5-R1	CTCCGCTCCTCCTCTCCTTGC<br>GCTGAATCCACAACCAAGTG	Amplifying *xa5* flanking PE target site
xa23p-F1<br>xa23HR-R8	CCCGAACATCACTAACATCG<br>TCATGTGTATACCGGCTACGG	Amplifying *xa23* flanking PE target site
OsEPSPS-F1<br>OsEPSPS-R1	GGCACTCCATCAAGCACATA<br>CTCGGGCTCTCTGTGGAAG	Amplifying *OsEPSPS1* flanking PE target site
OsALS-F1<br>OsALS-R1	AGAAGAGTGAAGTCCGTGCC<br>GGGTCATTCAGGTCAAACATAGG	Amplifying *OsALS1* flanking PE target site
OsSWT11-F10<br>OsSWT11-R10	ATTCAGAGTAGTGGAGAGAG<br>CTTCCTGACACAAGAGATGC	Amplifying *OsSWEET11a* flanking PE target site
OsGS2-F1<br>OsGS2-R1	GTACAGTTGGATATGGTCCG<br>ATCTTTGCCTGTTCCACCAC	Amplifying *OsGS2* flanking PE target site
OsSPL13-F1<br>OsSPL13-R1	CCCTTTCAACCTTTTCCACC<br>GAGCTAGTGCTACTGTGTGC	Amplifying *OsSPL13* flanking PE target site
OsSPL14-F1<br>OsSPL14-R1	AGTGCAGTGGCGGGATATGG<br>GGGGCTTGGTGCCATGTAGC	Amplifying *OsSPL14* flanking PE target site

xa23p-F3<br>Xa23HR-R11	CTCTTTCCCTACACGACgctcttccgatctTCGTCGTGGTGGTGAGATTAAG<br>ctggagttcagacgtgtgctcttccgatctTCATGTGTATACCGGCTACGG	Deep amplicon sequencing of *xa23* PE edits
Xa5-F1<br>Xa5-R2	CTCTTTCCCTACACGACgctcttccgatctCATTGGCATGTGCCTCACTG<br>ctggagttcagacgtgtgctcttccgatctCTCCAAGGCTTCCGTCATAG	Deep amplicon sequencing of *xa5* PE edits
OsSWT11-F11<br>OsSWT11-R11	CTCTTTCCCTACACGACgctcttccgatctATTCAGAGTAGTGGAGAGAG<br>ctggagttcagacgtgtgctcttccgatctCTTCCTGACACAAGAGATGC	Deep amplicon sequencing of *OsSWEET11a* PE edits
OsGS2-F2<br>OsGS2-R2	CTCTTTCCCTACACGACgctcttccgatctGTACAGTTGGATATGGTCCG<br>ctggagttcagacgtgtgctcttccgatctTAGCAGGGTAAAGAGAGTGG	Deep amplicon sequencing of *OsGS2* PE edits
OsSPL13-F2<br>OsSPL13-R2	CTCTTTCCCTACACGACgctcttccgatctCCCTTTCAACCTTTTCCACC<br>ctggagttcagacgtgtgctcttccgatctGAGCTAGTGCTACTGTGTGC	Deep amplicon sequencing of *OsSPL13* PE edits
xa23RT-F1<br>xa23RT-R1	TCTACCTCTGCCGCTTTCTC<br>GGGAGAATAACCATCTTGTCGTC	RT-PCR of *Xa23*
SWT11RTF1<br>SWT11RTR1	GGGATTTCTGGCTAGTTTCT<br>CGAGGTAGAGGACGATGTAG	RT-PCR of *OsSWEEt11*
OsActinF3<br>OsActinR3	CTCAGCACATTCCAGCAGAT<br>ACAGATAGGCCGGTTGAAAA	RT-PCR of *OsActin*
Truseq-R49<br>Truseq-R50<br>Truseq-R51<br>Truseq-R52<br>Truseq-R53<br>Truseq-R54<br>Truseq-R55<br>Truseq-R56<br>Truseq-R57<br>Truseq-R58	CAAGCAGAAGACGGCATACGAGAT<u>**CGAGTAAT**</u>GTGACTGGAGTTCAGACGTGTGCT<br>CAAGCAGAAGACGGCATACGAGAT<u>**TCTCCGGA**</u>GTGACTGGAGTTCAGACGTGTGCT<br>CAAGCAGAAGACGGCATACGAGAT<u>**AATGAGCG**</u>GTGACTGGAGTTCAGACGTGTGCT<br>CAAGCAGAAGACGGCATACGAGAT<u>**GGAATCTC**</u>GTGACTGGAGTTCAGACGTGTGCT<br>CAAGCAGAAGACGGCATACGAGAT<u>**TTCTGAAT**</u>GTGACTGGAGTTCAGACGTGTGCT<br>CAAGCAGAAGACGGCATACGAGAT<u>**ACGAATTC**</u>GTGACTGGAGTTCAGACGTGTGCT<br>CAAGCAGAAGACGGCATACGAGAT<u>**AGCTTCAG**</u>GTGACTGGAGTTCAGACGTGTGCT<br>CAAGCAGAAGACGGCATACGAGAT<u>**GCGCATTA**</u>GTGACTGGAGTTCAGACGTGTGCT<br>CAAGCAGAAGACGGCATACGAGAT<u>**CATAGCCG**</u>GTGACTGGAGTTCAGACGTGTGCT<br>CAAGCAGAAGACGGCATACGAGAT<u>**TTCGCGGA**</u>GTGACTGGAGTTCAGACGTGTGCT	Deep amplicon sequencing with dual multiple barcodes (bold and underlined)

Truseq-R59	CAAGCAGAAGACGGCATACGAGAT**GCGCGAGA**GTGACTGGAGTTCAGACGTGTGCT	
Truseq-R60	CAAGCAGAAGACGGCATACGAGAT**CTATCGCT**GTGACTGGAGTTCAGACGTGTGCT	
TruSeq-F1	AATGATACGGCGACCACCGAGATCTACAC**TATAGCCT**ACACTCTTTCCCTACACGACGCTCT	
TruSeq-F2	AATGATACGGCGACCACCGAGATCTACAC**ATAGAGGC**ACACTCTTTCCCTACACGACGCTCT	
TruSeq-F3	AATGATACGGCGACCACCGAGATCTACAC**CCTATCCT**ACACTCTTTCCCTACACGACGCTCT	
TruSeq-F4	AATGATACGGCGACCACCGAGATCTACAC**GGCTCTGA**ACACTCTTTCCCTACACGACGCTCT	
TruSeq-F5	AATGATACGGCGACCACCGAGATCTACAC**AGGCGAAG**ACACTCTTTCCCTACACGACGCTCT	
TruSeq-F6	AATGATACGGCGACCACCGAGATCTACAC**TAATCTTA**ACACTCTTTCCCTACACGACGCTCT	
TruSeq-F7	AATGATACGGCGACCACCGAGATCTACAC**CAGGACGT**ACACTCTTTCCCTACACGACGCTCT	
TruSeq-F8	AATGATACGGCGACCACCGAGATCTACAC**GTACTGAC**ACACTCTTTCCCTACACGACGCTCT	

Supplemental Table 2. Sequence related to pegRNAs and ngRNAs

Gene	Edit	pegRNA/ngRNA	Sequence
TFIIAγ5	V39E	target	AAGTAGATACCTTATCAAAC
		rtT/PBS/linker	GCCATTCAAGTTCTTGAGCAGTT/TGATAAGGTATC/AGGAATAA
		nicking gRNA	AGTTCTTGagCAGTTTGATA
xa23	EBE knock-in	target	GTAGCTGATGTTAGTGAGG
		rtT/PBS/linker	GCCTTCCTTCCGCCTTATATAAACCCCCTCCAACCAGGTGCTAAG/CACTAACATCA/AAATTCAT
		nicking gRNA	GTTCCTTGTTGCATCATCTCA
OsEPSPS1	TAP > IVS	target	GCAGTCACGGCTGCTGTCAA
		rtT/PBS/linker	TGGAAtTGtAATGCGAtCATTG/ACAGCAGCCGTGA/AGAAATAT
		nicking gRNA	TGTTGAGAAGGATGCGAAAG
OsALS1	S627I, G628G	target	GCTGCCTATGATCCCAAGTG
		rtT/PBS/linker	GCCgCCga/TTGGGATCATAGGC/TTAAACAG
		nicking gRNA	TCCTTGAATGCGCCgCCgaT
OsSWEET11a	EBE knock-out	target	CACTTTTGGTGGTGTACAGT
		rtT/PBS/linker	AAAGGTTAGATATGCATCTactagt/GTACACCACCAA/TTTATAAA
		nicking gRNA	CtagtGTACACCACCAAAAG
OsGS2	mir396 knock-out	target	GCGACCAGCTGCGTTTCCAC
		rtT/PBS/linker	ACCGCGGCCGCAACCGcagccGcAAGCCgGTc/GAAACGCAGC/ACATACAG
		nicking gRNA1	GCCGCAACCGcagccGcAAGC
		nicking gRNA2	GAAATGGCGGTGCTCGAAGG
OsSPL13	6-bp deletion, *Spe*I insertion	target	GCTCGAGCTCGAGCTCATGAG
		rtT/PBS/linker1	TCCTCCTCCGCCTTCCACTTCCACTC/ATGAGCTCGAGCT/AAATATAT
		nicking gRNA1	GCCACCGCTTCCACCTCCCCT
		rtT/PBS/linker2	CTTCagatctCACTTCCACTC/ATGAGCTCGAGCT/ATTAACTT
		nicking gRNA2	GCACCCTGCCTCCACACACA

OsSPL14	L292I, T297T	target	GGTAGTAGTATCCCATGGCT
		rtT/PBS/linker	gtgcTGTGCTaTCTCTCTTCTGTCAACaCAGC/CATGGGATACTA/AGGAATAA
		nicking gRNA	GCTGGCCCAAATCTCCCTCC

Supplemental Table 3. Deduced genotype of *xa5* and *Xa23*^{SWT11} duplex edited plants based on PCR-RE.

Line #	Deduced genotype	
	TFIIAγ5/xa5	*xa23/Xa23*^{SWT11}
2	Bi	Bi
3	Mo	Mo
7	Mo	Mo
11	WT	WT
14	Mo	De
17	WT	WT
18	WT	WT
19	Mo	Mo
20	Mo	WT
24	WT	WT
25	WT	WT
28	Mo	Mo
29	Mo	Mo
33	Mo	Mo
34	Bi	Bi
35	Bi	WT
37	W	WT
42	Mo	Mo
43	Mo	Mo
46	Bi	Mo
47	WT	WT
48	WT	Mo
49	WT	Mo
50	WT	Mo
51	WT	Mo
52	WT	WT

Mo: Monoallelic edit, Bi: Biallelic edit, WT: unedited wildtype, De: Deletion

Supplemental Table 4. Deduced genotype of *EPSPS1* (TAP > IVS) and *OsSWEET11a/xa13* duplex edited plants based on PCR-RE.

Line #	Deduced genotype	
	OsEPSPS1 (TAP > IVS)	*OsSWEET11a/xa13*
1	Mo	WT
2	Mo	Mo
3	Mo	Mo
4	Mo	Mo
5	Mo	Bi
6	Mo	WT
7	W	WT
8	Mo	Bi
9	WT	WT
10	Mo	Mo
11	Mo	Mo
12	Mo	Mo
13	Mo	WT
14	Mo	WT
15	WT	WT
16	WT	WT
17	Mo	Mo
18	Mo	Mo
19	WT	WT
20	Mo	Bi
21	Mo	Mo

Mo: Monoallelic edit, Bi: Biallelic edit, WT: unedited wildtype

Supplemental Table 5. Deduced genotype of *TFIIAγ5/xa5, OsEPSPS1* (TAP > IVS), *OsALS* (S627I) and *OsSWEET11a/xa13* quadruplex edited plants based on PCR-RE.

Line #	Deduced genotype			
	TFIIAγ5/xa5	*OsEPSPS1* (TAP > IVS)	*OsALS (S627I)*	*OsSWEET11a/xa13*
1	Mo	Mo	Mo	Mo
7	Mo	Mo	Bi	WT
12	Mo	Bi	Bi	Mo
15	Mo	Mo	Bi	Bi
19	WT	Mo	Mo	WT
20	Bi	Bi	Mo	Mo
21	Mo	Bi	Mo	Mo
22	WT	Mo	Mo	WT
28	Mo	Bi	Bi	Bi
29	Mo	Mo	Bi	Mo
30	WT	Mo	Mo	Mo
34	Mo	Mo	Mo	Mo
37	WT	Mo	Mo	WT
47	WT	Mo	Mo	WT
50	Mo	Mo	Mo	Mo
52	Mo	Mo	Mo	Bi
53	Mo	Bi	Mo	Bi
55	Mo	WT	Mo	WT
56	WT	Bi	WT	Mo
58	WT	Mo	Bi	Mo
59	Mo	Mo	Mo	Mo
62	Mo	WT	WT	WT
63	Mo	Mo	Mo	WT

Mo: Monoallelic edit, Bi: Biallelic edit, WT: unedited wildtype

Supplemental Table 6. Deduced genotype of *OsEPSPS1* (TAP > IVS), *TFIIAγ5/xa5,* and *OsSPL14/IPA1* triplex edited plants based on PCR-RE.

Line #	Deduced genotype		
	OsEPSPS1 (TAP > IVS)	*TFIIAγ5/xa5*	*OsSPL14/IPA1*
1	WT	WT	WT
2	WT	Mo	WT
3	WT	Mo	Mo
4	WT	Mo	WT
5	Mo	Mo	Bi
6	Mo	WT	Mo
7	WT	Mo	Mo
8	WT	Mo	Mo
9	WT	WT	WT
10	WT	WT	WT
11	WT	WT	WT
12	Mo	WT	Mo
13	WT	Mo	WT
14	WT	WT	Mo
15	Mo	Mo	WT
16	WT	WT	WT
17	Mo	WT	Mo

Mo: Monoallelic edit, Bi: Biallelic edit, WT: unedited wildtype

Supplemental Table 7. Editing rates of triplex prime editing in construct targeting *OsEPSPS1* (TAP > IVS), *TFIIAγ5/xa5,* and *OsSPL14/IPA1*.

	**# of genes edited**			
	**zero**	**one**	**two**	**three**
**# of T_0 Lines**	5 (29.4%)	4 (23.5%)	7 (41.1%)	1 (5.9%)

Supplemental Table 8. Deduced genotype of *OsGS2 (miR396-KO), TFIIAγ5/xa5,* and *OsSPL14/IPA1* triplex edited plants based on PCR-RE.

Line #	Deduced genotype		
	OsGS2 (miR396-KO)	*TFIIAγ5/xa5*	*OsSPL14/IPA1*
1	WT	WT	WT
2	WT	Mo	Mo
3	WT	WT	WT
4	WT	Mo	Mo
5	WT	WT	WT
6	WT	Mo	WT
7	WT	WT	Mo
8	WT	WT	WT
9	WT	Mo	Mo
10	WT	WT	WT
11	WT	WT	WT
12	WT	WT	WT
13	WT	WT	WT
14	WT	Mo	Mo
15	WT	WT	WT
16	WT	Mo	WT
17	WT	Mo	Mo
18	WT	WT	WT
19	WT	WT	Mo
20	WT	WT	WT
21	WT	WT	WT
22	WT	WT	Mo
23	WT	WT	Bi
24	WT	WT	WT
25	WT	Mo	Mo

Mo: Monoallelic edit, Bi: Biallelic edit, WT: unedited wildtype

Supplemental Table 9. Editing rates of triplex prime editing in construct targeting *OsGS2 (miR396-KO), TFIIAγ5/xa5,* and *OsSPL14/IPA1*.

	**# of genes edited**			
	**zero**	**one**	**two**	**three**
**# of T_0 Lines**	13 (52%)	6 (24%)	6 (24%)	0 (0.0%)

Supplemental Table 10. Deduced genotype of *OsGS2 (miR396-KO)*, *OsSPL13/OsGLW7*, *TFIIAγ5/xa5,* and *OsSPL14/IPA1* quadruplex edited plants based on PCR-RE.

Line #	Deduced genotype			
	OsGS2 (miR396-KO)-1	*SPL13/GLW7-1*	*TFIIAγ5/xa5*	*OsSPL14/IPA1*
1	WT	WT	Mo	WT
4	WT	WT	Mo	WT
5	WT	WT	WT	Mo
7	WT	WT	WT	Mo
8	WT	WT	WT	Mo
11	WT	WT	WT	WT
12	WT	WT	Mo	WT
14	WT	WT	WT	WT
16	WT	WT	WT	Mo
19	WT	WT	Mo	WT
22	WT	WT	Mo	Bi
24	WT	WT	Mo	Mo
25	WT	WT	Mo	WT
26	WT	WT	WT	WT
27	WT	WT	WT	Bi
28	WT	WT	Mo	Mo
29	WT	WT	WT	Mo
30	WT	WT	WT	WT
31	WT	WT	Mo	Mo
33	WT	WT	Mo	WT
34	WT	WT	WT	WT
35	WT	WT	Mo	WT
36	WT	WT	Mo	Mo

Mo: Monoallelic edit, Bi: Biallelic edit, WT: unedited wildtype

Supplemental Table 11. Editing rates of quadruplex prime editing in construct targeting *OsGS2 (miR396-KO), OsSPL13/OsGLW7, TFIIAγ5/xa5,* and *OsSPL14/IPA1*.

	**# of genes edited**				
	**zero**	**one**	**two**	**three**	**four**
**# of T_0 Lines**	5 (21.7%)	13 (56.5%)	5 (21.7%)	0 (0.0%)	0 (0.0%)

Supplemental Table 12. Deduced genotype of *OsGS2 (miR396-KO)-2*, *OsSPL13/OsGLW7-2*, *TFIIAγ5/xa5,* and *OsSPL14/IPA1* quadruplex edited plants based on PCR-RE.

Line #	Deduced genotype			
	OsGS2 (miR396-KO)-2	*OsSPL13/OsGLW7-2*	*TFIIAγ5/xa5*	*OsSPL14/IPA1*
1	WT	WT	Mo	Mo
2	WT	WT	Mo	Mo
3	WT	WT	WT	WT
4	WT	Mo	WT	WT
5	WT	WT	WT	WT
6	WT	WT	Mo	Mo
7	WT	WT	WT	WT
8	WT	WT	Mo	Mo
9	WT	WT	WT	WT
10	WT	WT	Mo	WT
11	WT	WT	WT	WT
12	WT	WT	WT	Mo
13	WT	WT	WT	Mo
14	WT	WT	Mo	Mo
15	WT	Mo	WT	Mo
16	WT	Mo	Mo	Mo
17	WT	WT	Bi	WT
18	WT	Mo	WT	WT
19	WT	Mo	WT	WT
20	WT	WT	Mo	WT

Mo: Monoallelic edit, Bi: Biallelic edit, WT: unedited wildtype

Supplemental Table 13. Editing rates of quadruplex prime editing in construct targeting *OsGS2 (miR396-KO)-2*, *OsSPL13/GLW7-2, TFIIAγ5/xa5,* and *OsSPL14/IPA1*.

	# of genes edited				
	**zero**	**one**	**two**	**three**	**four**
**# of T_0 Lines**	5 (25%)	8 (40%)	6 (30%)	1 (5.0%)	0 (0.0%)

Supplemental Table 14. Deduced genotype of *OsEPSPS1 (TAP-IVS)*, *SPL13/GLW7-2*, *TFIIAγ5/xa5,* and *OsSPL14/IPA1* quadruplex edited plants based on PCR-RE.

Line #	Deduced genotype			
	OsEPSPS1 (TAP-IVS)	*OsSPL13/GLW7-2*	*TFIIAγ5/xa5*	*OsSPL14/IPA1*
1	Mo	WT	WT	WT
2	Bi	Mo	Mo	WT
3	Mo	WT	WT	WT
4	WT	WT	WT	WT
5	Mo	WT	WT	WT
6	Bi	Mo	WT	WT
7	WT	WT	Mo	Bi
8	WT	WT	Bi	WT
9	Mo	WT	WT	WT
10	Mo	WT	WT	WT
11	Mo	Bi	WT	WT
12	Mo	WT	WT	WT
13	Mo	WT	WT	WT
14	WT	WT	WT	WT
15	WT	WT	WT	WT
16	Bi	WT	Mo	Mo
17	Mo	WT	WT	WT
18	Mo	WT	WT	Mo

Mo: Monoallelic edit, Bi: Biallelic edit, WT: unedited wildtype

Supplemental Table 15. Editing rates of quadruplex prime editing in construct targeting *OsEPSPS1* (TAP-IVS), *OsSPL13/GLW7-2, TFIIAγ5/xa5,* and *OsSPL14/IPA1*.

	# of genes edited				
	**zero**	**one**	**two**	**three**	**four**
**# of T_0 Lines**	3 (16.7%)	9 (50%)	4 (22.22%)	2 (11.11%)	0 (0.0%)

Supplemental Sequences

Supplemental Sequence 1. Sequences of various *att*L and *att*R units used in this study.
>*att*L1
CAAATAATGATTTTATTTTGACTGATAGTGACCTGTTCGTTGCAACAAATTGATGAG
CAATGCTTTTTTATAATGCCAACTTTGTACAAAAAAGCAGGCT

Supplemental Sequence 2
>*att*L2
ACCCAGCTTTCTTGTACAAAGTTGGCATTATAAAAAATAATTGCTCATCAATTTGTTG
CAACGAACAGGTCACTATCAGTCAAAATAAAATCATTATTTC

Supplemental Sequence 3
>*att*L3
AAATAATGATTTTATTTTGACTGATAGTGACCTGTTCGTTGCAACAAATTGATGAGC
AATGCTTTTTTATAATGCCAACTTTGTATAATAAAGTTG

Supplemental Sequence 4
>*att*L4
CAACTTTTCTATACAAAGTTGGCATTATAAGAAAGCATTGCTTATCAATTTGTTGCA
ACGAACAGGTCACTATCAGTCAAAATAAAATCATTATTT

Supplemental Sequence 5
>*att*L5
AAATAATGATTTTATTTTGACTGATAGTGACCTGTTCGTTGCAACAAATTGATGAGC
AATGCTTTTTTATAATGCCAACTTTGTATACAAAAGTTG

Supplemental Sequence 6
>*att*R1
ACAAGTTTGTACAAAAAAGCTGAACGAGAAACGTAAAATGATATAAATATCAATAT
ATTAAATTAGATTTTGCATAAAAAACAGACTACATAATACTGTAAAACACAACATAT
CCAGTCATATTG

Supplemental Sequence 7
>*att*R2
CATAGTGACTGGATATGTTGTGTTTTACAGCATTATGTAGTCTGTTTTTTATGCAAAA
TCTAATTTAATATATTGATATTTATATCATTTTACGTTTCTCGTTCAGCTTTCTTGTAC
AAAGTGGT

Supplemental Sequence 8
>*att*R3
CAACTTTGTATAATAAAGTTGAACGAGAAACGTAAAATGATATAAATATCAATATAT
TAAATTAGATTTTGCATAAAAAACAGACTACATAATACTGTAAAACACAACATATCC
AGTCACTATG

Supplemental Sequence 9
>*att*R4
CATAGTGACTGGATATGTTGTGTTTTACAGTATTATGTAGTCTGTTTTTTATGCAAAA
TCTAATTTAATATATTGATATTTATATCATTTTACGTTTCTCGTTCAACTTTTCTATAC
AAAGTTGG

Supplemental Sequence 10

>*att*R5
CAACTTTGTATACAAAAGTTGAACGAGAAACGTAAAATGATATAAATATCAATATA
TTAAATTAGATTTTGCATAAAAAACAGACTACATAATACTGTAAAACACAACATATC
CAGTCACTATG

Supplemental Sequence 11. Full plasmid sequence of CmYCLVp-epegRNA entry vector used in this study (color coded).

*aat*LR-35Sen-CmYCLVp-AtU6p-tRNAgly-pegRNA-EvopreQ1-HDV-tRNAmet-ngRNAsc-polyT-*att*LR
NNNNNNNNNNNNNNNNNNNNNNNNNNNNNNNNNNNNNNNNNNNNNNNNNNNNNN
NNNNNNNNNNNNNNNNNNNNNNNNNNNNNNNNNNNNNNNNNNNNNNCCGAATTA
TGGAGTCAAAGATTCAAATAGAGGACCTAACAGAACTCGCCGTAAAGACTGGCGAA
CAGTTCATACAGAGTCTCTTACGACTCAATGACAAGAAGAAAATCTTCGTCAACATG
GTGGAGCACGACACACTTGTCTACTCCAAAAATATCAAAGATACAGTCTCAGAAGA
CCAAAGGGCAATTGAGACTTTTCAACAAAGGGTAATATCCGGAAACCTCCTCGGATT
CCATTGCCCAGCTATCTGTCACTTTATTGTGAAGATAGTGGAAAAGGAAGGTGGCTC
CTACAAATGCCATCATTGCGATAAAGGAAAGGCCATCGTTGAAGATGCCTCTGCCG
ACAGTGGTCCCAAAGATGGACCCCCACCCACGAGGAGCATCGTGGAAAAAGAAGA
CGTTCCAACCACGTCTTCAAAGCAAGTGGATTGATGTGATTGGCAGACATACTGTCC
CACAAATGAAGATGGAATCTGTAAAAGAAAACGCGTGAAATAATGCGTCTGACAAA
GGTTAGGTCGGCTGCCTTTAATCAATACCAAAGTGGTCCCTACCACGATGGAAAAAC
TGTGCAGTCGGTTTGGCTTTTTCTGACGAACAAATAAGATTCGTGGCCGACAGGTGG
GGGTCCACCATGTGAAGGCATCTTCAGACTCCAATAATGGAGCAATGACGTAAGGG
CTTACGAAATAAGTAAGGGTAGTTTGGGAAATGTCCACTCACCCGTCAGTCTATAAA
TACTTAGCCCCTCCCTCATTGTTAAGGGAGCAAAATCTCAGAGAGATAGTCCTAGAG
AGAGAAAGAGAGCAAGTAGCCTAGAAGTAGTCAAGGCGGCGAAGTATTCAGGCAC
GTGGCCAGGAAGAAGAAAAGCCAAGACGACGAAAACAGGTAAGAGCTAAGCATCT
AGAAAGTTGAAAACAATCTTCAAAAGTCCCACATCGCTTAGATAAGAAAACGAAGC
TGAGTTTATATACAGCTAGAGTCGAAGTAGTGATTGAACAAAGCACCAGTGGTCTA
GTGGTAGAATAGTACCCTGCCACGGTACAGACCCGGGTTCGATTCCCGGCTGGTGCA
NNNNNNNNNNNNNNNNNNNGTTTAAGAGCTATGCTGGAAACAGCATAGCAAGTTT
AAATAAGGCTAGTCCGTTATCAACTTGAAAAAGTGGCACCGAGTCGGTGCNNNNNN
NNNNNNNNNNNNNNNNNNNNNNNNCGCGGTTCTATCTAGTTACGCGTTAAACCAAC
TAGAAAGGCCGGCATGGTCCCAGCCTCCTCGCTGGCGCCGGCTGGGCAACATGCTTC
GGCATGGCGAATGGGACAACAACAAATCAGAGTGGCGCAGCGGAAGCGTGGTGGG
CCCATAACCCACAGGTCCCAGGATCGAAACCTGGCTCTGATANNNNNNNNNNNNNN
NNNNNGTTTTAGAGCTAGAAATAGCAAGTTAAAATAAGGCTAGTCCGTTATCAACTT
GAAAAAGTGGCACCGAGTCGGTGCTTTTTTTTTGGCCGGCATGGTCCCAGCCTCCTC
GCTGGCGCCGGCTGGGCAACATGCTTCGGCATGGCGAATGGGACTTTTTTTTTGATA
TCTCCGGGGCTAATTGAATATGAAGATGAAGATGAAATATTTGGTGTGTCAAATAAA
AAGCTGGTGTGCTTAAGTTTGTGTTTTTTTCTTGGCTTGTTGTGTTATGAATTTGTGGC
TTTTTCTAATATTAAATGAATGTAAGATCTCATTATAATGAATAAACAAATGTTTCTA
TAATCCATTGTGAATGTTTTGTTGGATCTCTTCTGCAGCATATAACTACTGTATGTGC
TATGGTATGGACTATGGAATATGATTAAAGATAAGGAATTCGNNNNNNNNNNNNNN

NNNNNNNNNNNNNNNNNNNNNNNNNNNNNNNNNNNNNNNNNNNNNNNNNNNNNNN
NNNNNNNNNNNNNNNNNNNNNNNNNNNNNNNNN

Supplemental Sequence 12. Full plasmid sequence of pG3H-PE3max-attR1R2 used in this study (color coded).

attR1-ccdB-attR2-Zm*Ubi* promoter-bpNLS[SV40]-nCas9 (H840A)-linker-M-MLV RT-NLS[SV40]-NLS[c-myc]-E9 terminator-CaMV 35S promoter-Hygromycin-CaMV terminator

GTTTACCCGCCAATATATCCTGTCAAACACTGATAGTTTAAAGCTAgagacGGGGATCA
CAAGTTTGTACAAAAAAGCTGAACGAGAAACGTAAAATGATATAAATATCAATATA
TTAAATTAGATTTTGCATAAAAAACAGACTACATAATACTGTAAAACACAACATATC
CAGTCATATTGGCGGCCGCATTAGGCACCCCAGGCTTTACACTTTATGCTTCCGGCT
CGTATAATGTGTGGATTTTGAGTTAGgatcGATCCGGCTTACTAAAAGCCAGATAACA
GTATGCGTATTTGCGCGCTGATTTTTGCGGTATAAGAATATATACTGATATGTATACC
CGAAGTATGTCAAAAAGAGGTATGCTATGAAGCAGCGTATTACAGTGACAGTTGAC
AGCGACAGCTATCAGTTGCTCAAGGCATATATGATGTCAATATCTCCGGTCTGGTAA
GCACAACCATGCAGAATGAAGCCCGTCGTCTGCGTGCCGAACGCTGGAAAGCGGAA
AATCAGGAAGGGATGGCTGAGGTCGCCCGGTTTATTGAAATGAACGGCTCTTTTGCT
GACGAGAACAGGGGCTGGTGAAATGCAGTTTAAGGTTTACACCTATAAAAGAGAGA
GCCGTTATCGTCTGTTTGTGGATGTACAGAGTGATATTATTGACACGCCCGGGCGAC
GGATGGTGATCCCCCTGGCCAGTGCACGTCTGCTGTCAGATAAAGTCTCCCGTGAAC
TTTACCCGGTGGTGCATATCGGGGATGAAAGCTGGCGCATGATGACCACCGATATG
GCCAGTGTGCCGGTCTCCGTTATCGGGGAAGAAGTGGCTGATCTCAGCCACCGCGA
AAATGACATCAAAAACGCCATTAACCTGATGTTCTGGGGAATATAAATGTCAGGCTC
CCTTATACACAGCCAGTCTGCAGGTCGACCATAGTGACTGGATATGTTGTGTTTTAC
AGCATTATGTAGTCTGTTTTTTATGCAAAATCTAATTTAATATATTGATATTTATATC
ATTTTACGTTTCTCGTTCAGCTTTCTTGTACAAAGTGGTGCTTAAGTTTGTGTTTTTTT
CTTGGCTTGTTGTGTTATGAATTTGTGGCTTTTTCTAATATTAAATGAATGTAAGATC
TCATTATAATGAATAAACAAATGTTTCTATAATCCATTGTGAATGTTTTGTTGGATCT
CTTCTGCAGCATATAACTACTGTATGTGCTATGGTATGGACTATGGAATATGATTAA
AGATAACTAGTGCAGCGTGACCCGGTCGTGCCCCTCACTAGAGATAATGAGCATTGC
ATGTCTAAGTTATAAAAAATTACCACATATTTTTTTTGTCACACTTGTTTGAAGTGCA
GTTTATCTATCTTTATACATATATTTAAACTTTACTCTACGAATAATATAATCTATAG
TACTACAATAATATCAGTGTTTTAGAGAATCATATAAATGAACAGTTAGACATGGTC
TAAAGGACAATTGAGTATTTTGACAACAGGACTCTACAGTTTTATCTTTTTAGTGTGC
ATGTGTTCTCCTTTTTTTTTGCAAATAGCTTCACCTATATAATACTTCATCCATTTTAT
TAGTACATCCATTTAGGGTTTAGGGTTAATGGTTTTTATAGACTAATTTTTTTAGTAC
ATCTATTTTATTCTATTTTAGCCTCTAAATTAAGAAAACTAAAACTCTATTTTAGTTT
TTTTATTTAATAATTTAGATATAAAATAGAATAAAATAAAGTGACTAAAAATTAAAC
AAATACCCTTTAAGAAATTAAAAAAACTAAGGAAACATTTTTCTTGTTTCGAGTAGA
TAATGCCAGCCTGTTAAACGCCGTCGACGAGTCTAACGGACACCAACCAGCGAACC
AGCAGCGTCGCGTCGGGCCAAGCGAAGCAGACGGCACGGCATCTCTGTCGCTGCCT
CTGGACCCCTCTCGAGAGTTCCGCTCCACCGTTGGACTTGCTCCGCTGTCGGCATCC
AGAAATTGCGTGGCGGAGCGGCAGACGTGAGCCGGCACGGCAGGCGGCCTCCTCCT
CCTCTCACGGCACCGGCAGCTACGGGGGATTCCTTTCCCACCGCTCCTTCGCTTTCCC

TTCCTCGCCCGCCGTAATAAATAGACACCCCCTCCACACCCTCTTTCCCCAACCTCGT
GTTGTTCGGAGCGCACACACACACAACCAGATCTCCCCCAAATCCACCCGTCGGCAC
CTCCGCTTCAAGGTACGCCGCTCGTCCTCCCCCCCCCCCCCTCTCTACCTTCTCTAGA
TCGGCGTTCCGGTCCATGCTTAGGGCCCGGTAGTTCTACTTCTGTTCATGTTTGTGTT
AGATCCGTGTTTGTGTTAGATCCGTGCTGCTAGCGTTCGTACACGGATGCGACCTGT
ACGTCAGACACGTTCTGATTGCTAACTTGCCAGTGTTTCTCTTTGGGGAATCCTGGG
ATGGCTCTAGCCGTTCCGCAGACGGGATCGATTTCATGATTTTTTTTGTTTCGTTGCA
TAGGGTTTGGTTTGCCCTTTTCCTTTATTTCAATATATGCCGTGCACTTGTTTGTCGGG
TCATCTTTTCATGCTTTTTTTTGTCTTGGTTGTGATGATGTGGTCTGGTTGGGCGGTCG
TTCTAGATCGGAGTACAATTCTGTTTCAAACTACCTGGTGGATTTATTAATTTTGGAT
CTGTATGTGTGTGCCATACATATTCATAGTTACGAATTGAAGATGATGGATGGAAAT
ATCGATCTAGGATAGGTATACATGTTGATGCGGGTTTTACTGATGCATATACAGAGA
TGCTTTTTGTTCGCTTGGTTGTGATGATGTGGTGTGGTTGGGCGGTCGTTCATTCGTT
CTAGATCGGAGTAGAATACTGTTTCAAACTACCTGGTGTATTTATTAATTTTGGAACT
GTATGTGTGTGTCATACATCTTCATAGTTACGAGTTTAAGATGGATGGAAATATCGA
TCTAGGATAGGTATACATGTTGATGTGGGTTTTACTGATGCATATACATGATGGCAT
ATGCAGCATCTATTCATATGCTCTAACCTTGAGTACCTATCTATTATAATAAACAAGT
ATGTTTTATAATTATTTTGATCTTGATATACTTGGATGATGGCATATGCAGCAGCTAT
ATGTGGATTTTTTTAGCCCTGCCTTCATACGCTATTTATTTGCTTGGTACTGTTTCTTT
TGTCGATGCTCACCCTGTTGTTTGGTGTTACTTCTGCAGGTACCTAGGCCTCTAGATG
AAGAGGACAGCCGATGGCAGCGAGTTCGAGAGCCCTAAGAAGAAGAGGAAGGTGG
ACAAGAAGTACTCGATCGGCCTCGATATTGGGACTAACTCTGTTGGCTGGGCCGTGA
TCACCGACGAGTACAAGGTGCCCTCAAAGAAGTTCAAGGTCCTGGGCAACACCGAT
CGGCATTCCATCAAGAAGAATCTCATTGGCGCTCTCCTGTTCGACAGCGGCGAGACG
GCTGAGGCTACGCGGCTCAAGCGCACCGCCCGCAGGCGGTACACGCGCAGGAAGAA
TCGCATCTGCTACCTGCAGGAGATTTTCTCCAACGAGATGGCGAAGGTTGACGATTC
TTTCTTCCACAGGCTGGAGGAGTCATTCCTCGTGGAGGAGGATAAGAAGCACGAGC
GGCATCCAATCTTCGGCAACATTGTCGACGAGGTTGCCTACCACGAGAAGTACCCTA
CGATCTACCATCTGCGGAAGAAGCTCGTGGACTCCACAGATAAGGCGGACCTCCGC
CTGATCTACCTCGCTCTGGCCCACATGATTAAGTTCAGGGGCCATTTCCTGATCGAG
GGGGATCTCAACCCGGACAATAGCGATGTTGACAAGCTGTTCATCCAGCTCGTGCAG
ACGTACAACCAGCTCTTCGAGGAGAACCCCATTAATGCGTCAGGCGTCGACGCGAA
GGCTATCCTGTCCGCTAGGCTCTCGAAGTCTCGGAAGCTCGAGAACCTGATCGCCCA
GCTGCCGGGCGAGAAGAAGAACGGCCTGTTCGGGAATCTCATTGCGCTCAGCCTGG
GGCTCACGCCCAACTTCAAGTCGAATTTCGATCTCGCTGAGGACGCCAAGCTGCAGC
TCTCCAAGGACACATACGACGATGACCTGGATAACCTCCTGGCCCAGATCGGCGAT
CAGTACGCGGACCTGTTCCTCGCTGCCAAGAATCTGTCGGACGCCATCCTCCTGTCT
GATATTCTCAGGGTGAACACCGAGATTACGAAGGCTCCGCTCTCAGCCTCCATGATC
AAGCGCTACGACGAGCACCATCAGGATCTGACCCTCCTGAAGGCGCTGGTCAGGCA
GCAGCTCCCCGAGAAGTACAAGGAGATCTTCTTCGATCAGTCGAAGAACGGCTACG
CTGGGTACATTGACGGCGGGGCCTCTCAGGAGGAGTTCTACAAGTTCATCAAGCCG
ATTCTGGAGAAGATGGACGGCACGGAGGAGCTGCTGGTGAAGCTCAAGCGCGAGGA
CCTCCTGAGGAAGCAGCGGACATTCGATAACGGCAGCATCCCACACCAGATTCATC
TCGGGGAGCTGCACGCTATCCTGAGGAGGCAGGAGGACTTCTACCCTTTCCTCAAGG
ATAACCGCGAGAAGATCGAGAAGATTCTGACTTTCAGGATCCCGTACTACGTCGGCC
CACTCGCTAGGGGCAACTCCCGCTTCGCTTGGATGACCCGCAAGTCAGAGGAGACG

ATCACGCCGTGGAACTTCGAGGAGGTGGTCGACAAGGGCGCTAGCGCTCAGTCGTT
CATCGAGAGGATGACGAATTTCGACAAGAACCTGCCAAATGAGAAGGTGCTCCCTA
AGCACTCGCTCCTGTACGAGTACTTCACAGTCTACAACGAGCTGACTAAGGTGAAGT
ATGTGACCGAGGGCATGAGGAAGCCGGCTTTCCTGTCTGGGGAGCAGAAGAAGGCC
ATCGTGGACCTCCTGTTCAAGACCAACCGGAAGGTCACGGTTAAGCAGCTCAAGGA
GGACTACTTCAAGAAGATTGAGTGCTTCGATTCGGTCGAGATCTCTGGCGTTGAGGA
CCGCTTCAACGCCTCCCTGGGGACCTACCACGATCTCCTGAAGATCATTAAGGATAA
GGACTTCCTGGACAACGAGGAGAATGAGGATATCCTCGAGGACATTGTGCTGACAC
TCACTCTGTTCGAGGACCGGGAGATGATCGAGGAGCGCCTGAAGACTTACGCCCAT
CTCTTCGATGACAAGGTCATGAAGCAGCTCAAGAGGAGGAGGTACACCGGCTGGGG
GAGGCTGAGCAGGAAGCTCATCAACGGCATTCGGGACAAGCAGTCCGGGAAGACG
ATCCTCGACTTCCTGAAGAGCGATGGCTTCGCGAACCGCAATTTCATGCAGCTGATT
CACGATGACAGCCTCACATTCAAGGAGGATATCCAGAAGGCTCAGGTGAGCGGCCA
GGGGGACTCGCTGCACGAGCATATCGCGAACCTCGCTGGCTCGCCAGCTATCAAGA
AGGGGATTCTGCAGACCGTGAAGGTTGTGGACGAGCTGGTGAAGGTCATGGGCAGG
CACAAGCCTGAGAACATCGTCATTGAGATGGCCCGGGAGAATCAGACCACGCAGAA
GGGCCAGAAGAACTCACGCGAGAGGATGAAGAGGATCGAGGAGGGCATTAAGGAG
CTGGGGTCCCAGATCCTCAAGGAGCACCCGGTGGAGAACACGCAGCTGCAGAATGA
GAAGCTCTACCTGTACTACCTCCAGAATGGCCGCGATATGTATGTGGACCAGGAGCT
GGATATTAACAGGCTCAGCGATTACGACGTCGATGCCATCGTTCCACAGTCATTCCT
GAAGGATGACTCCATTGACAACAAGGTCCTCACCAGGTCGGACAAGAACCGGGGCA
AGTCTGATAATGTTCCTTCAGAGGAGGTCGTTAAGAAGATGAAGAACTACTGGCGC
CAGCTCCTGAATGCCAAGCTGATCACGCAGCGGAAGTTCGATAACCTCACAAAGGC
TGAGAGGGGCGGGCTCTCTGAGCTGGACAAGGCGGGCTTCATCAAGAGGCAGCTGG
TCGAGACACGGCAGATCACTAAGCACGTTGCGCAGATTCTCGACTCACGGATGAAC
ACTAAGTACGATGAGAATGACAAGCTGATCCGCGAGGTGAAGGTCATCACCCTGAA
GTCAAAGCTCGTCTCCGACTTCAGGAAGGATTTCCAGTTCTACAAGGTTCGGGAGAT
CAACAATTACCACCATGCCCATGACGCGTACCTGAACGCGGTGGTCGGCACAGCTCT
GATCAAGAAGTACCCAAAGCTCGAGAGCGAGTTCGTGTACGGGGACTACAAGGTTT
ACGATGTGAGGAAGATGATCGCCAAGTCGGAGCAGGAGATTGGCAAGGCTACCGCC
AAGTACTTCTTCTACTCTAACATTATGAATTTCTTCAAGACAGAGATCACTCTGGCCA
ATGGCGAGATCCGGAAGCGCCCCCTCATCGAGACGAACGGCGAGACGGGGGAGAT
CGTGTGGGACAAGGGCAGGGATTTCGCGACCGTCAGGAAGGTTCTCTCCATGCCAC
AAGTGAATATCGTCAAGAAGACAGAGGTCCAGACTGGCGGGTTCTCTAAGGAGTCA
ATTCTGCCTAAGCGGAACAGCGACAAGCTCATCGCCCGCAAGAAGGACTGGGATCC
GAAGAAGTACGGCGGGTTCGACAGCCCCACTGTGGCCTACTCGGTCCTGGTTGTGGC
GAAGGTTGAGAAGGGCAAGTCCAAGAAGCTCAAGAGCGTGAAGGAGCTGCTGGGG
ATCACGATTATGGAGCGCTCCAGCTTCGAGAAGAACCCGATCGATTTCCTGGAGGCG
AAGGGCTACAAGGAGGTGAAGAAGGACCTGATCATTAAGCTCCCCAAGTACTCACT
CTTCGAGCTGGAGAACGGCAGGAAGCGGATGCTGGCTTCCGCTGGCGAGCTGCAGA
AGGGGAACGAGCTGGCTCTGCCGTCCAAGTATGTGAACTTCCTCTACCTGGCCTCCC
ACTACGAGAAGCTCAAGGGCAGCCCCGAGGACAACGAGCAGAAGCAGCTGTTCGTC
GAGCAGCACAAGCATTACCTCGACGAGATCATTGAGCAGATTTCCGAGTTCTCCAAG
CGCGTGATCCTGGCCGACGCGAATCTGGATAAGGTCCTCTCCGCGTACAACAAGCA
CCGCGACAAGCCAATCAGGGAGCAGGCTGAGAATATCATTCATCTCTTCACCCTGAC
GAACCTCGGCGCCCCTGCTGCTTTCAAGTACTTCGACACAACTATCGATCGCAAGAG

GTACACAAGCACTAAGGAGGTCCTGGACGCGACCCTCATCCACCAGTCGATTACCG
GCCTCTACGAGACGCGCATCGACCTGTCTCAGCTCGGGGGCGACTCAGGCGGCTCAT
CTGGCGGGTCAAAGCGCACAGCCGACGGCTCTGAGTTCGAGAGCCCTAAGAAGAAG
CGCAAGGTGTCAGGCGGCTCTTCAGGCGGCAGCACCCTGAACATTGAGGACGAGTA
CCGGCTGCACGAGACGAGCAAGGAGCCAGACGTTTCGCTCGGCAGCACTTGGCTCT
CTGACTTCCCACAGGCTTGGGCCGAGACTGGCGGCATGGGCCTGGCCGTGCGCCAG
GCTCCACTGATCATCCCTCTGAAGGCGACCTCCACCCCGGTTTCTATTAAGCAGTAC
CCGATGAGCCAGGAGGCCAGGCTGGGGATCAAGCCACACATTCAGCGGCTGCTGGA
CCAGGGCATCCTGGTGCCATGCCAGTCCCCGTGGAATACTCCGCTCCTGCCGGTGAA
GAAGCCTGGGACAAACGACTACAGGCCGGTTCAGGATCTCAGGGAGGTGAACAAGC
GCGTGGAGGACATCCATCCGACAGTGCCGAACCCGTACAATCTGCTGTCGGGCCTG
CCTCCGAGCCACCAGTGGTACACCGTCCTGGACCTCAAGGACGCTTTCTTCTGCCTG
CGGCTGCACCCGACGTCTCAGCCGCTGTTCGCGTTCGAGTGGCGCGACCCAGAGATG
GGCATTTCCGGCCAGCTGACCTGGACACGCCTACCCCAGGGCTTCAAGAACTCCCCG
ACTCTCTTCAACGAGGCTCTCCACCGGGATCTCGCGGACTTCAGGATTCAGCATCCC
GATCTGATCCTGCTCCAGTATGTTGACGACCTCCTCCTGGCCGCGACGTCGGAGCTG
GACTGCCAGCAGGGCACCCGGGCGCTGCTGCAGACACTGGGCAATCTGGGGTACCG
CGCCTCTGCGAAGAAGGCGCAGATCTGCCAGAAGCAAGTGAAGTACCTGGGCTACC
TCCTGAAGGAGGGCCAGCGCTGGCTCACTGAGGCGAGGAAGGAGACTGTTATGGGC
CAGCCCACTCCAAAGACTCCGAGGCAGCTCAGGGAGTTCCTCGGCAAGGCTGGGTT
CTGCCGCCTGTTCATCCCTGGGTTCGCTGAGATGGCTGCGCCGCTCTACCCGCTGAC
TAAGCCGGGGACACTGTTCAACTGGGGGCCAGACCAGCAGAAGGCGTACCAGGAG
ATTAAGCAGGCGCTGCTGACGGCCCCAGCGCTCGGCCTACCAGACCTGACGAAGCC
GTTCGAGCTGTTCGTTGACGAGAAGCAGGGGTACGCGAAGGGCGTGCTGACACAGA
AGCTGGGGCCTTGGCGCCGCCCGGTCGCGTACCTGTCGAAGAAGCTGGACCCAGTC
GCTGCTGGGTGGCCTCCATGCCTCCGGATGGTCGCTGCTATTGCGGTTCTGACCAAG
GATGCGGGGAAGCTCACAATGGGGCAGCCTCTCGTGATCCTGGCTCCACATGCGGT
GGAGGCGCTGGTGAAGCAGCCACCGGACCGGTGGCTGTCGAACGCTCGGATGACAC
ACTACCAGGCGCTCCTCCTCGATACAGACCGGGTTCAGTTCGGGCCTGTGGTTGCTC
TGAACCCAGCCACACTGCTGCCACTCCCTGAGGAGGGCCTCCAGCACAATTGCCTCG
ACATCCTGGCTGAGGCGCACGGCACCCGCCCTGATCTCACCGACCAGCCTCTGCCAG
ATGCTGACCACACCTGGTACACGGATGGGTCCTCGCTGCTGCAGGAGGGCCAGAGG
AAGGCGGGCGCCGCCGTCACCACAGAGACAGAGGTTATTTGGGCCAAGGCCCTACC
GGCTGGCACCAGCGCCCAGCGCGCTGAGCTGATCGCGCTGACTCAGGCGCTGAAGA
TGGCCGAGGGGAAGAAGCTCAATGTTTACACCGACTCGCGGTACGCGTTCGCTACA
GCTCACATTCATGGGGAGATCTACCGCCGGCGCGGGTGGCTGACTTCGGAGGGCAA
GGAGATTAAGAATAAGGACGAGATCCTGGCCCTGCTCAAGGCGCTGTTCCTGCCGA
AGCGCCTCTCAATCATTCACTGCCCGGGCCACCAGAAGGGCCATTCGGCCGAGGCT
AGGGGCAATCGGATGGCTGACCAGGCGGCGCGGAAGGCGGCTATCACCGAGACTCC
CGATACATCTACCCTCCTGATCGAGAACTCGAGCCCATCTGGCGGCTCTAAGCGGAC
TGCGGATGGGTCTGAGTTCGAGTCACCAAAGAAGAAGAGGAAGGTGGGCTCTGGCC
CTGCTGCTAAGCGCGTGAAGCTCGATTGAGCTCAGAGCTTTCGTTCGTATCATCGGT
TTCGACAACGTTCGTCAAGTTCAATGCATCAGTTTCATTGCGCACACACCAGAATCC
TACTGAGTTTGAGTATTATGGCATTGGGAAAACTGTTTTTCTTGTACCATTTGTTGTG
CTTGTAATTTACTGTGTTTTTTATTCGGTTTTCGCTATCGAACTGTGAAATGGAAATG
GATGGAGAAGAGTTAATGAATGATATGGTCCTTTTGTTCATTCTCAAATTAATATTA

TTTGTTTTTCTCTTATTTGTTGTGTGTTGAATTTGAAATTATAAGAGATATGCAAAC
ATTTTGTTTTGAGTAAAAATGTGTCAAATCGTGGCCTCTAATGACCGAAGTTAATAT
GAGGAGTAAAACACTTGTAGTTGTACCATTATGCTTATTCACTAGGCAACAAATATA
TTTTCAGACCTAGAAAAGCTGCAAATGTTACTGAATACAAGTATGTCCTCTTGTGTTT
TAGACATTTATGAACTTTCCTTTATGTAATTTTCCAGAATCCTTGTCAGATTCTAATC
ATTGCTTTATAATTATAGTTATACTCATGGATTTGTAGTTGAGTATGAAAATATTTTT
TAATGCATTTTATGACTTGCCAATTGATTGACAACGAATTCGCTACCTTAGGACCGTT
ATAGTTACGTTAATTAAGAATTATCGAACCACTTTGTACAAGAAAGCTGGGTCTAGC
TGGCGCGCCATATAAGCTTATTACCCTGTTATCCCTAatcgatcgatcgGTTAACGGTAACC
GACTTGCTGCCCCGAGAATTATGCAGCATTTTTTTGGTGTATGTGGGCCCCAAATGA
AGTGCAGGTCAAACCTTGACAGTGACGACAAATCGTTGGGCGGGTCCAGGGCGAAT
TTTGCGACAACATGTCGAGGCTCAGCAGGACGAATTCGTAACTATAACGGTCCTAAG
GTAGCGAATTAATTAACATGATTACGCCAAGCTATCAACTTTGGTATAGAAAAGTTG
TTAGAATTGGCGCGAATTCGTAATCATGTCATAGCTGTTTCCTGTGTGAAATTGTTAT
CCGCTCACAATTCCACACAACATACGAGCCGGAAGCATAAAGTGTAAAGCCTGGGG
TGCCTAATGAGTGAGCTAACTCACATTAATTGCGTTGCGCTCACTGCCCGCTTTCCA
GTCGGGAAACCTGTCGTGCCAGCTGCATTAATGAATCGGCCAACGCGCGGGGAGAG
GCGGTTTGCGTATTGGCTAGAGCAGCTTGCCAACATGGTGGAGCACGACACTCTCGT
CTACTCCAAGAATATCAAAGATACAGTCTCAGAAGACCAAAGGGCTATTGAGACTT
TTCAACAAAGGGTAATATCGGGAAACCTCCTCGGATTCCATTGCCCAGCTATCTGTC
ACTTCATCAAAAGGACAGTAGAAAAGGAAGGTGGCACCTACAAATGCCATCATTGC
GATAAAGGAAAGGCTATCGTTCAAGATGCCTCTGCCGACAGTGGTCCCAAAGATGG
ACCCCCACCCACGAGGAGCATCGTGGAAAAAGAAGACGTTCCAACCACGTCTTCAA
AGCAAGTGGATTGATGTGAACATGGTGGAGCACGACACTCTCGTCTACTCCAAGAA
TATCAAAGATACAGTCTCAGAAGACCAAAGGGCTATTGAGACTTTTCAACAAAGGG
TAATATCGGGAAACCTCCTCGGATTCCATTGCCCAGCTATCTGTCACTTCATCAAAA
GGACAGTAGAAAAGGAAGGTGGCACCTACAAATGCCATCATTGCGATAAAGGAAA
GGCTATCGTTCAAGATGCCTCTGCCGACAGTGGTCCCAAAGATGGACCCCCACCCAC
GAGGAGCATCGTGGAAAAAGAAGACGTTCCAACCACGTCTTCAAAGCAAGTGGATT
GATGTGATATCTCCACTGACGTAAGGGATGACGCACAATCCCACTATCCTTCGCAAG
ACCCTTCCTCTATATAAGGAAGTTCATTTCATTTGGAGAGGACACGCTGAAATCACC
AGTCTCTCTCTACAAATCTATCTCTCTCGAGCTTTCGCAGATCCGGGGGGCAATGAG
ATATGAAAAAGCCTGAACTCACCGCGACGTCTGTCGAGAAGTTTCTGATCGAAAAG
TTCGACAGCGTCTCCGACCTGATGCAGCTCTCGGAGGGCGAAGAATCTCGTGCTTTC
AGCTTCGATGTAGGAGGGCGTGGATATGTCCTGCGGGTAAATAGCTGCGCCGATGG
TTTCTACAAAGATCGTTATGTTTATCGGCACTTTGCATCGGCCGCGCTCCCGATTCCG
GAAGTGCTTGACATTGGGGAGTTTAGCGAGAGCCTGACCTATTGCATCTCCCGCCGT
TCACAGGGTGTCACGTTGCAAGACCTGCCTGAAACCGAACTGCCCGCTGTTCTACAA
CCGGTCGCGGAGGCTATGGATGCGATCGCTGCGGCCGATCTTAGCCAGACGAGCGG
GTTCGGCCCATTCGGACCGCAAGGAATCGGTCAATACACTACATGGCGTGATTTCAT
ATGCGCGATTGCTGATCCCCATGTGTATCACTGGCAAACTGTGATGGACGACACCGT
CAGTGCGTCCGTCGCGCAGGCTCTCGATGAGCTGATGCTTTGGGCCGAGGACTGCCC
CGAAGTCCGGCACCTCGTGCACGCGGATTTCGGCTCCAACAATGTCCTGACGGACA
ATGGCCGCATAACAGCGGTCATTGACTGGAGCGAGGCGATGTTCGGGGATTCCCAA
TACGAGGTCGCCAACATCTTCTTCTGGAGGCCGTGGTTGGCTTGTATGGAGCAGCAG
ACGCGCTACTTCGAGCGGAGGCATCCGGAGCTTGCAGGATCGCCACGACTCCGGGC

GTATATGCTCCGCATTGGTCTTGACCAACTCTATCAGAGCTTGGTTGACGGCAATTTC
GATGATGCAGCTTGGGCGCAGGGTCGATGCGACGCAATCGTCCGATCCGGAGCCGG
GACTGTCGGGCGTACACAAATCGCCCGCAGAAGCGCGGCCGTCTGGACCGATGGCT
GTGTAGAAGTACTCGCCGATAGTGGAAACCGACGCCCCAGCACTCGTCCGAGGGCA
AAGAAATAGAGTAGATGCCGACCGGGATCTGTCGATCGACAAGCTCGAGTTTCTCC
ATAATAATGTGTGAGTAGTTCCCAGATAAGGGAATTAGGGTTCCTATAGGGTTTCGC
TCATGTGTTGAGCATATAAGAAACCCTTAGTATGTATTTGTATTTGTAAAATACTTCT
ATCAATAAAATTTCTAATTCCTAAAACCAAAATCCAGTACTAAAATCCAGATCCCCC
GAATTAATTCGGCGTTAATTCAGTACATTAAAAACGTCCGCAATGTGTTATTAAGTT
GTCTAAGCGTCAATTTGTTTACACCACAATATATCCTGCCACCAGCCAGCCAACAGC
TCCCCGACCGGCAGCTCGGCACAAAATCACCACTCGATACAGGCAGCCCATCAGTC
CGGGACGGCGTCAGCGGGAGAGCCGTTGTAAGGCGGCAGACTTTGCTCATGTTACC
GATGCTATTCGGAAGAACGGCAACTAAGCTGCCGGGTTTGAAACACGGATGATCTC
GCGGAGGGTAGCATGTTGATTGTAACGATGACAGAGCGTTGCTGCCTGTGATCACCG
CGGTTTCAAGCTTGTATTCACCGGTGTTATGGTTAATTAACAGCTTCAACTTTATTAT
ACAAAGTTGATAATTCACTGGCGCGCCGTAACTATAACGGTCCTAAGGTAGCGAAC
CGCGGCTGGCAAACAGCTATTATGGGTATTATGGGTCTAGTGGGTTTCAAAATCGGC
TCCGTCGATACTATGTTATACGCCAACTTTGAAAACAACTTTGAAAAAGCTGTTTTCT
GGTATTTAAGGTTTTAGAATGCAAGGAACAGTGAATTGGAGTTCGTCTTGTTATAAT
TAGCTTCTTGGGGTATCTTTAAATACTGTAGAAAAGAGGAAGGAAATAATAAATGG
CTAAAATGAGAATATCACCGGAATTGAAAAAACTGATCGAAAAATACCGCTGCGTA
AAAGATACGGAAGGAATGTCTCCTGCTAAGGTATATAAGCTGGTGGGAGAAAATGA
AAACCTATATTTAAAAATGACGGACAGCCGGTATAAAGGGACCACCTATGATGTGG
AACGGGAAAAGGACATGATGCTATGGCTGGAAGGAAAGCTGCCTGTTCCAAAGGTC
CTGCACTTTGAACGGCATGATGGCTGGAGCAATCTGCTCATGAGTGAGGCCGATGGC
GTCCTTTGCTCGGAAGAGTATGAAGATGAACAAAGCCCTGAAAAGATTATCGAGCT
GTATGCGGAGTGCATCAGGCTCTTTCACTCCATCGACATATCGGATTGTCCCTATAC
GAATAGCTTAGACAGCCGCTTAGCCGAATTGGATTACTTACTGAATAACGATCTGGC
CGATGTGGATTGCGAAAACTGGGAAGAAGACACTCCATTTAAAGATCCGCGCGAGC
TGTATGATTTTTTAAAGACGGAAAAGCCCGAAGAGGAACTTGTCTTTTCCCACGGCG
ACCTGGGAGACAGCAACATCTTTGTGAAAGATGGCAAAGTAAGTGGCTTTATTGATC
TTGGGAGAAGCGGCAGGGCGGACAAGTGGTATGACATTGCCTTCTGCGTCCGGTCG
ATCAGGGAGGATATCGGGGAAGAACAGTATGTCGAGCTATTTTTTGACTTACTGGGG
ATCAAGCCTGATTGGGAGAAAATAAAATATTATATTTTACTGGATGAATTGTTTTAG
TACCTAATCTCGGGGACGTCTAACTACTAAGCGAGAGTAGGGAACTGCCAGGCATC
AAATAAAACGAAAGGCTCAGTCGGAAGACTGGGCCTTTCGTTTTATCTGTTGTTTGT
CGGTGAACGCTCTCCTGAGTAGGACAAATCCGCCGGGAGCGGATTTGAACGTTGTG
AAGCAACGGCCCGGAGGGTGGCGGGCAGGACGCCCGCCATAAACTGCCAGGCATC
AAACTAAGCAGAAGGCCATCCTGACGGATGGCCTTTTTGCGTTTCTACAAACTCTTC
CTGTTAGATGCATGACCAAAATCCCTTAACGTGAGTTTTCGTTCCACTGAGCGTCAG
ACCCCGTAGAAAAGATCAAAGGATCTTCTTGAGATCCTTTTTTTCTGCGCGTAATCT
GCTGCTTGCAAACAAAAAAACCACCGCTACCAGCGGTGGTTTGTTTGCCGGATCAAG
AGCTACCAACTCTTTTTCCGAAGGTAACTGGCTTCAGCAGAGCGCAGATACCAAATA
CTGTCCTTCTAGTGTAGCCGTAGTTAGGCCACCACTTCAAGAACTCTGTAGCACCGC
CTACATACCTCGCTCTGCTAATCCTGTTACCAGTGGCTGCTGCCAGTGGCGATAAGT
CGTGTCTTACCGGGTTGGACTCAAGACGATAGTTACCGGATAAGGCGCAGCGGTCG

GGCTGAACGGGGGGTTCGTGCACACAGCCCAGCTTGGAGCGAACGACCTACACCGA
ACTGAGATACCTACAGCGTGAGCTATGAGAAAGCGCCACGCTTCCCGAAGGGAGAA
AGGCGGACAGGTATCCGGTAAGCGGCAGGGTCGGAACAGGAGAGCGCACGAGGGA
GCTTCCAGGGGGAAACGCCTGGTATCTTTATAGTCCTGTCGGGTTTCGCCACCTCTG
ACTTGAGCGTCGATTTTTGTGATGCTCGTCAGGGGGGCGGAGCCTATGGAAAAACGC
CAGCAACGCGGCCTTTTTACGGTTCCTGGCCTTTTGCTGGCCTTTTGCTCACATGTTC
TTTCCTGCGTTATCCCCTGATTCTGTGGATAACCGTATTACCGCTAGATAGCTGGTTG
CCCTCGCCGCTGGGCTGGCGGCCGTCTATGGCCCTGCAAACGCGCCAGAAACGCCG
TCGAAGCCGTGTGCGAGACACCCCGGCCGCCGGCGTTGTGGATACCTCGCGGAAAA
CTTGGCCCTCACTGACAGATGAGGGGCGGACGTTGACACTTGAGGGGCCGACTCAC
CCGGCGCGGCGTTGACAGATGAGGGGCAGGCTCGATTTCGGCCGGCGACGTGGAGC
TGGCCAGCCTCGCAAATCGGCGAAAACGCCTGATTTTACGCGAGTTTCCCACAGATG
ATGTGGACAAGCCTGGGGATAAGTGCCCTGCGGTATTGACACTTGAGGGGCGCGAC
TACTGACAGATGAGGGGCGCGATCCTTGACACTTGAGGGGCAGAGTGCTGACAGAT
GAGGGGCGCACCTATTGACATTTGAGGGGCTGTCCACAGGCAGAAAATCCAGCATT
TGCAAGGGTTTCCGCCCGTTTTTCGGCCACCGCTAACCTGTCTTTTAACCTGCTTTTA
AACCAATATTTATAAACCTTGTTTTTAACCAGGGCTGCGCCCTGTGCGCGTGACCGC
GCACGCCGAAGGGGGGTGCCCCCCCTTCTCGAACCCTCCCGGTCGAGTCATGGTCTA
GCATGGATCTCGGGGACGTCACCCATAATACCCATAATAGCTGTTTGCCAGTACTTT
GATCCCGAGGGGAACCCTGTGGTTGGCATGCACATACAAATGGACGAACGGATAAA
CCTTTTCACGCCCTTTTAAATATCCGTTATTCTAATAAACGCTCTTTTCTCTTAG

Supplemental protocol.

pegRNA-ngRNA design and PE clone construction.

1. Prime edit gRNA (pegRNA) has three components; spacer for targeting, extension for priming and reverse transcription template, and nick gRNA for nicking the un-edited strand.
    a. To design a spacer, the nearest PAM that is NGG (for sense strand) or CCN (for anti-sense strand) sequence to the site for editing needs to be identified.
    b. As the nCas9 cutting site is between 3rd and 4th nucleotide upstream of PAM, the edit has to lie before the cutting site in case of CCN PAM and after the cutting site in case of NGG PAM. The spacer should be 20-nt long and design the oligoes like this for cloning into entry vector;
        Forward oligo: tgcaNNNNNNNNNNNNNNNNNNNN
        Reverse oligo: aaacNNNNNNNNNNNNNNNNNNNN
    c. For NGG PAM, the forward oligo is exaclty same as that of the 5' to 3' target strand and for CCN PAM, the forward oligo is reverse complementary to the 5' to 3' target strand.
    To design extension oligos, two websites can be used. First is, http://www.plantgenomeediting.net. This will help find the best template and primer binding site (PBS) (Default parameters can be used for designing template and PBS and recommended sequences can be used for designing constructs). Second is, https://peglit.liugroup.us, this one is needed for designing linker between the PBS and pseudoknot (The scaffold sequence needs to be changed to

this engineered guide RNA scaffold: "GTTTAAGAGCTATGCTGGAAACAGCATAGCAAGTTTAAATAAGGCTAGTCCGTTATCAACTTGAAAAAGTGGCACCGAGTCGGTGC" before designing linker in pegLIT website).

d. Finally the forward extension oligo should look like this;

gtgc[recommended template][recommended PBS][Linker]

For example,
extension F: gtgctcaagttcttgGAcagtttgataaggtatcCAGAAGAC
extension R: cgcgGTCTTCTGgataccttatcaaactgTCcaagaacttga

e. Nick gRNA (ngRNA) design: For nick gRNA design, please follow two rules:
   i. If spacer PAM is NGG nick PAM sould be CCN or vice-versa. This means nick and spacer should target opposite strand.
   ii. Pick a nick gRNA within -100 to +100 bp of the edit site.
   iii. Any gRNA designing program such as CHOPCHOP can be used to find the best ngRNA within -100 to +100 bp of the edit site.
f. The nick oligo should look like this
   Nick forward: gataNNNNNNNNNNNNNNNNNNNN
   Nick reverse: aaacNNNNNNNNNNNNNNNNNNNN

2. Cloning of spacer, extension and nick in entry vector
   a. The spacer is cloned at *Bsm*BI sites followed by extension and nick cloning at *Bsa*I sites.
      i. For cloning spacer:
      Digest entry vector with *BsmB*I, after purification ligate the digested vector with annealed forward and reverse oligos and transform *E. coli* cells. For screening, use spacer reverse oligo combined to yCLV-F1 primer 5'-TAGCCTAGAAGTAGTCAAGG-3' for colony PCR.
      ii. For cloning extension and nick gRNA:
      Golden gate using *Bsa*I on spacer ligated entry vector, individually annealed extension and nick oligos and T4 ligase in T4 buffer followed by transformation of *E. coli* cells. The fragment between extension and nick gRNA is not supplied separately and the endogenous fragment released upon *Bsa*I digestion is sufficient to complete the cloning process. For screening, use spacer forward and nick reverse oligo to do colony PCR followed by *XcmI/AflII* digestion of plasmid for final confirmation.
      iii. For sequencing, use yCLV-F1 primer 5'-TAGCCTAGAAGTAGTCAAGG-3` primer.
3. Gateway of spacer, extenison and nick ligated entry vector/vectors into PEmax-GW destination vector.
   a. Use LR clonase II to mobilize the pegRNA-ngRNA region of entry vector into PEmax-GW and transform *E. coli* cells. For screening use *Mlu*I for digestion of plasmid extracted from single colonies.
4. Trasnformation of *Agrobacterium* with binary vector PE3max-pegRNA-ngRNA.

a. Transform the *Agrobacterium* strain carrying the helper plasmid pVIS-VIR2. We use LBA4404/pVIS-VIR2 in our experiments. Screen the colonies using spacer forward oligo and nick reverse oligo.

Design considerations for PE3 editing: Here we list few considerations when designing editing experiments using PE3:

1. The closest spacer sequence to the edit should be preferred as the pegRNA, however considerations for farther spacers can be taken if the closer spacer has problems like higher off-targets, poor GC content or secondary structures. Software such as CHOPCHOP can be used to select the high efficiency spacers near the edit site.
2. We design our nick gRNA within 100-bp to the edit site either upstream or downstream. CHOPCHOP can be used to find the high efficiency nick gRNA spacers.
3. Nick can be designed to target only the edited strand by using spacer spanning the edited site, only if possible. This is termed as PE3b system and it is shown to decrease the occurrences of deletions due to double strand breaks. However, caution should be taken to avoid transgene targeting by nick gRNA.
4. The PBS length can be calculated using the http://www.plantgenomeediting.net webtool. This tool takes care of the annealing temperature. The length of template can be variable and in our experiments, we use the rule of termination for rtT. In rule of termination, we stop the rtT before a “C”, “GC”, or “TGC” to avoid the gRNA scaffold templated by-products. For example, if the gDNA has “ATTTCT**TGC**AGTCGTAGCAAA” then our rtT will stop before “TGC” and it would look like “AGTCGTAGCAAA”. “TGC” is best case scenario followed by “GC” and “C” respectively.
